# Sleep loss leads to the withdrawal of human helping across individuals, groups, and large-scale societies

**DOI:** 10.1371/journal.pbio.3001733

**Published:** 2022-08-23

**Authors:** Eti Ben Simon, Raphael Vallat, Aubrey Rossi, Matthew P. Walker

**Affiliations:** 1 Center for Human Sleep Science, Department of Psychology, University of California, Berkeley, California, United States of America; 2 Helen Wills Neuroscience Institute, University of California, Berkeley, California, United States of America; Oxford University, UNITED KINGDOM

## Abstract

Humans help each other. This fundamental feature of homo sapiens has been one of the most powerful forces sculpting the advent of modern civilizations. But what determines whether humans choose to help one another? Across 3 replicating studies, here, we demonstrate that sleep loss represents one previously unrecognized factor dictating whether humans choose to help each other, observed at 3 different scales (within individuals, across individuals, and across societies). First, at an individual level, 1 night of sleep loss triggers the withdrawal of help from one individual to another. Moreover, fMRI findings revealed that the withdrawal of human helping is associated with deactivation of key nodes within the social cognition brain network that facilitates prosociality. Second, at a group level, ecological night-to-night reductions in sleep across several nights predict corresponding next-day reductions in the choice to help others during day-to-day interactions. Third, at a large-scale national level, we demonstrate that 1 h of lost sleep opportunity, inflicted by the transition to Daylight Saving Time, reduces real-world altruistic helping through the act of donation giving, established through the analysis of over 3 million charitable donations. Therefore, inadequate sleep represents a significant influential force determining whether humans choose to help one another, observable across micro- and macroscopic levels of civilized interaction. The implications of this effect may be non-trivial when considering the essentiality of human helping in the maintenance of cooperative, civil society, combined with the reported decline in sufficient sleep in many first-world nations.

## Abstract 2

Helping behavior between humans has been one of the most influential forces sculpting modern civilizations, but what factors influence this propensity to help? This study demonstrates that a lack of sleep dictates whether humans choose to help each other at three different scales: within individuals, across individuals, and across societies.

“*Service to others is the rent you pay for your room here on earth*.”

― **Muhammad Ali**

Humans help each other. Helping is a prominent feature of homo sapiens [1], and represents a fundamental force sculpting the advent and preservation of modern civilizations [2,3].

The ubiquity of helping is evident across the full spectrum of societal strata. From global government-to-government aid packages (e.g., the international aid following the 2004 Indian Ocean tsunami [4]), to country-wide pledge drives (e.g., the 2010 Haiti disaster) [5], and to individuals altruistically gifting money or donating their own blood to strangers, the expression of helping is abundant and pervasive [6]. So much so that this fundamental act has scaled into a lucent and sizable “helping economy” [7], with charitable giving in the United States amounting to $450 billion in 2019; a value representing 5.5% of the gross domestic product. In the United Kingdom, 10 billion pounds were donated to charity in 2017 and 2018. Indeed, more than 50% of individuals across the US, Europe, and Asia will have reported donating to charity or helping a stranger within the past month (The World Giving index).

Human helping is therefore globally abundant, common across diverse societies, sizable in scope, substantive in financial magnitude, consequential in ramification, and frequent in occurrence.

The motivated drive for humans to help each other has been linked to a range of underlying factors, from evolutionary forces (e.g., kin selection and reciprocal altruism that bias helping toward close others [2]), cultural norms and expectations (e.g., individualistic versus collectivistic cultures [8,9]), to socioeconomic factors (e.g., helping is less common in larger cities relative to rural areas [10,11]), as well as personality traits (e.g., individual empathy) [12,13].

Ultimately, however, the decisional act to help others involves the human brain. Prosocial helping of varied kinds consistently engages a set of brain regions known as the social cognition network. Comprised of the medial prefrontal cortex (mPFC), mid and superior temporal sulcus, temporal-parietal junction (TPJ), and the precuneus [14,15], this network is activated when considering the mental states, needs, and perspectives of others [16–19], and the active choice to help them [20–23]. In contrast, lesions within key regions of this network result in “acquired sociopathy” [24], associated with a loss of both empathy and the withdrawal of compassionate helping [25–27].

Yet the possibility that sleep loss represents another significant factor determining whether or not humans help each other, linked to underlying impairments within the social cognition brain network, remains unknown. Several lines of evidence motivate this prediction. First, insufficient sleep impairs emotional processing, including deficits in emotion recognition and expression, while conversely increasing basic emotional reactivity, further linked to antisocial behavior [28,29] (such as increased interpersonal conflict [30] and reduced trust in others [31,32]). Second, sleep loss reliably decreases activity in, and disrupts functional connectivity between, numerous regions within the social cognition brain network [33], including the mPFC [34], TPJ, and precuneus [35].

Building on this overarching hypothesis, here, we test the prediction that a lack of sleep impairs human helping at a neural, individual, group, and global societal level. More specifically, we tested whether: (i) within individuals, a night of experimental sleep loss decreases the fundamental desire to help others, the underlying neural mechanism of which is linked to impaired activity within the social cognition brain network when considering other individuals (Study 1), (ii) in a micro-longitudinal study, night-to-night fluctuations in sleep result in a corresponding next-day deficit in the desire to act altruistically and helping others (Study 2), and (iii) at a large-scale national level, the loss of 1 h of sleep opportunity, using the manipulation of daylight saving time (DST), impairs the real-world behavioral act of altruistic human helping at a large-scale, societal level (Study 3).

## Results

In short (but see Methods for details), Study 1 involved 24 healthy adult participants taking part in a counterbalanced, cross-over experimental design with two conditions: one night of sleep, and one night of sleep deprivation. In each condition, participants performed a standardized helping questionnaire as well as a social cognition task performed during a functional MRI scan. Study 2 involved a microlongitudinal design evaluating a total of 136 individuals. Participants completed helping questionnaires and sleep diaries for 4 consecutive days under free-living conditions. Finally, Study 3 assessed large-scale altruistic donation behavior during the annual transition to DST, analyzing over 3 million charitable donations made between the years 2001 to 2016 in the US.

**Fig 1:**
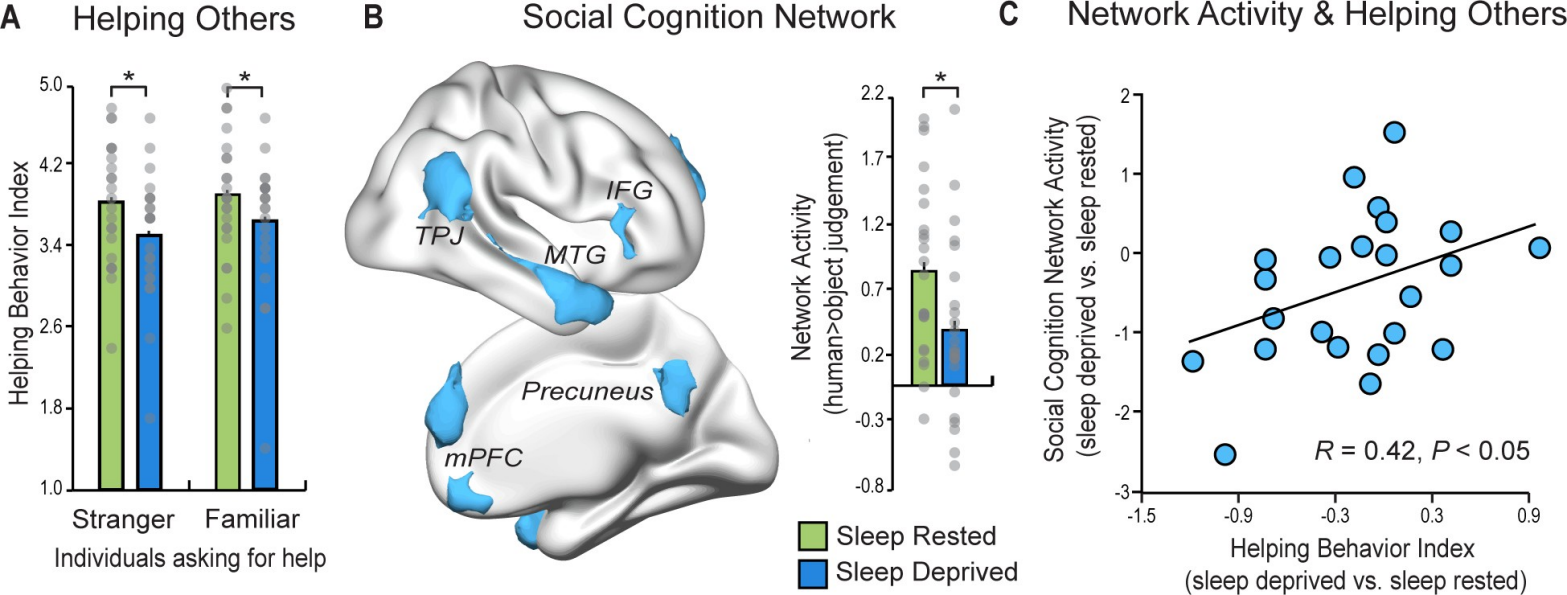
In-laboratory Study 1. (**A**) One night of sleep deprivation was associated with a significant decrease in helping desire, relative to the sleep-rested condition, for both circumstances involving strangers (left) and familiar others (right). (**B**) Activity in the social cognition network (left, meta-analysis-based activation mask, corrected for multiple comparisons, *P*_FDR_ < 0.01) was significantly reduced following sleep deprivation, relative to the sleep-rested condition (right). (**C**) The relative reduction in social cognition brain network activity under conditions of sleep loss was significantly associated with lower helping behavior across individuals. *P < 0.05; error bars reflect standard error of the mean. Individual data presented in this figure can be found in S1 Data. IFG, inferior frontal gyrus; mPFC, medial prefrontal cortex; MTG, middle temporal gyrus; TPJ, temporal-parietal junction.

Consistent with the first hypothesis, participants in Study 1 demonstrated a significant decrease in the desire to help others under conditions of sleep deprivation, relative to those same individuals when sleep rested (*N* = 23, helping behavior score (means ± SE): SR = 3.88 ± 0.12, SD = 3.59 ± 0.13, main effect of sleep F_(1,22)_ = 7.67, η_p_^2^ = 0.259, *d* = 1.054, mean difference = −0.29 ± 0.1, 95% CI = [−0.07, −0.51], *P* = 0.011, see **Fig 1** and S1 Data).

This effect of sleep loss was also consistent across participants, such that 78% of individuals demonstrated a reduction in the desire to help others. The deficit in helping following sleep loss further remained significant when controlling for individual changes in mood, as well as changes in task-assessed motivational effort (*β* = *−0*.*55* ± 0.18, *t = −2*.*9*, *P* < *0*.*01*, R^2^ = 0.28, see Methods for respective measures). Moreover, the sleep loss impairment in helping was not significantly related to trait levels of empathy (R = 0.3, *P* > 0.15). Therefore, the detrimental impact of insufficient sleep on the prosocial act of helping does not appear to be parsimoniously accounted for by changes in mood state, the willingness to exert effort, or individual empathy.

Interestingly, and perhaps unexpectedly considering theories of kin selection [2,36], the withdrawal of helping caused by sleep loss was significant no matter whether the circumstance involved helping a stranger or helping someone familiar (i.e., friends/colleagues) (Strangers: *t*_(22)_ = −2.47, *P* = 0.021; Familiar others: *t*_(22)_ = −2.66, *P* = 0.014), with neither being significantly different to the other in terms of the negative impact of sleep loss (interaction of familiarity and sleep, *F*_(1,22)_ = 0.65, *P* = 0.43, η_p_^2^ = 0.029, main effect of familiarity F_(1,22)_ = 1.76, η_p_^2^ = 0.074, *P* = 0.198, see Note A in S1 Text for additional familiarity analyses). Thus the impact of sleep loss on helping behavior is common across different conspecific contexts. This would suggest that interpersonal familiarity with the individual in need of help (e.g., a friend versus stranger) does not appear to confer immunity against the sleep loss-associated reduction in the desire to act altruistically, suggesting a broad, indiscriminate, impact of sleep loss on prosocial behavior, one that is not confined to specific contexts.

Next, we examined the underlying neural changes associated with the reduction in helping choices triggered by sleep deprivation. Functional MRI analyses focused a priori on the social cognition network, given its recognized involvement in prosocial behaviors [17,18,20,22,37]. Supporting the hypothesis, sleep loss was associated with a significant reduction in task-evoked activity within the social cognition network, relative to the sleep-rested condition (mean change ± SE = −0.46 ± 0.18, *P* = 0.02, d = 0.7, see Fig 1B). Furthermore, the magnitude of activity impairment in the social cognition network caused by sleep loss predicted the corresponding decrease in the desire to help others across participants, such that the greater the regional brain impairment, the greater the reduction in helping when sleep deprived (R = 0.42, *P* = 0.046, *n* = 23, see Fig 1C).

Of note, these effects were specific to the social cognition brain network—no other standard functional brain network demonstrated an association with either sleep loss or helping behavior (all *P* > 0.1 FDR-corrected for multiple comparisons, see Methods and Table A in S1 Text*)*. There was also no association between changes in helping and activity within the salience network, known to support empathy and social-emotional functioning [37–39]. Moreover, the observed changes in social cognition network activity following sleep deprivation were not associated with, nor best accounted for, by changes in attention and effort (Rs < 0.2, *P* > 0.3, see Methods for additional sensitivity analyses), as well as trait empathy scores (R = 0.18, *P* > 0.4). Finally, sleep loss-related changes in social cognition network activity remained significant when accounting for changes in positive and negative mood in the sleep loss condition (*β* = *−*0.78 ± 0.31, *P < 0*.*05*).

Testing the second experimental prediction, Study 2 sought to determine whether ecologically modest night-to-night variations in sleep, beyond experimental sleep loss, result in consequential next-day changes in the desire to help others, tracked across numerous consecutive days within the same individuals (*N* = 136, 441 observations). Here, self-reported sleep duration and sleep efficiency were analyzed across individuals (i.e., mean sleep duration/efficiency over the study period, the between-person effect), as well as within individuals, taking into account each person’s deviation from their own average. Study 2, therefore, differs from Study 1 in that Study 1 involved laboratory-controlled sleep manipulations that were causal (sleep versus no sleep), while Study 2 represents a paradigm more akin to free-living conditions. Specifically, Study 2 involved people adhering to their own sleep schedule and amount of sleep without any experimental manipulation.

**Fig 2:**
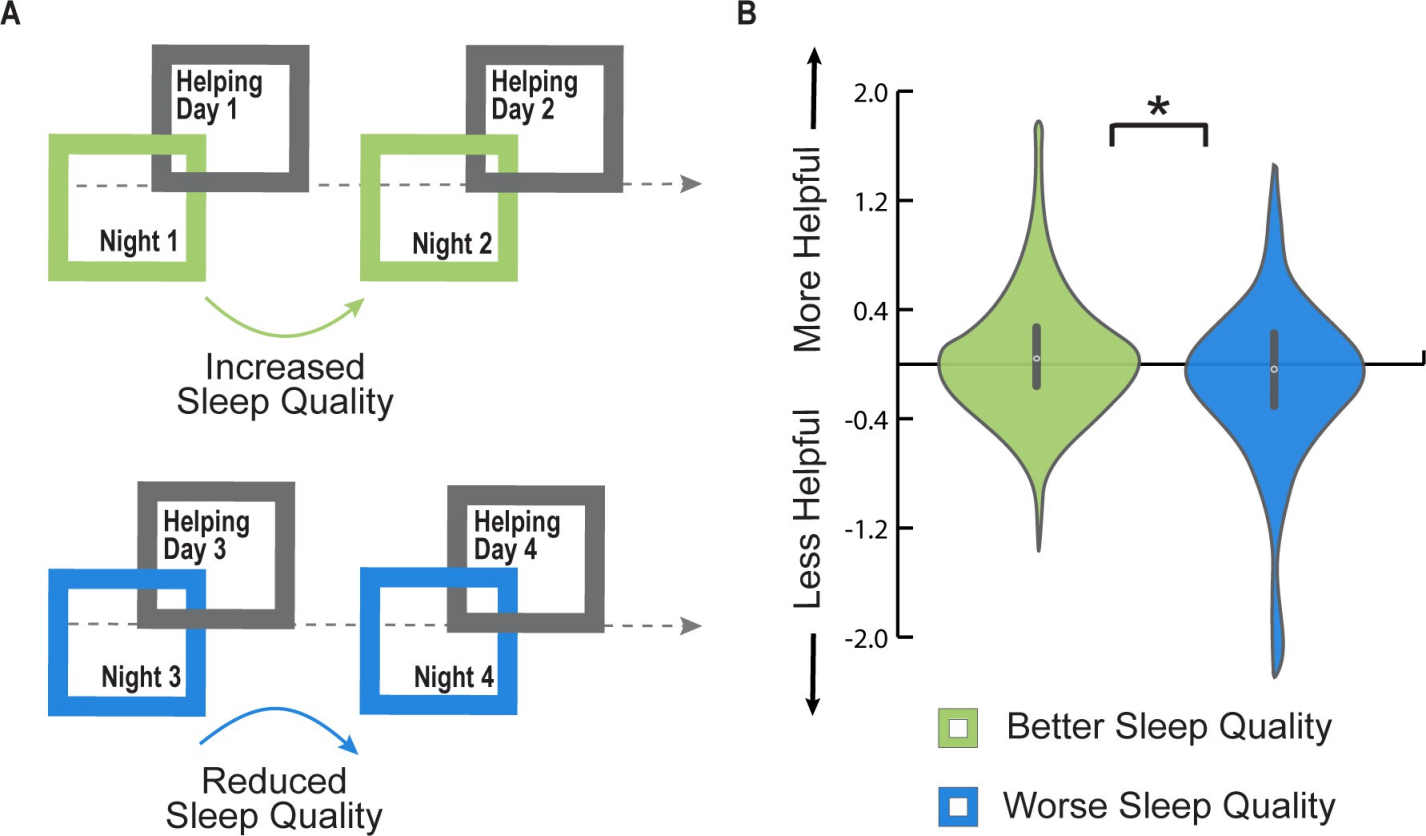
Micro-longitudinal Study 2. (**A**) Study design. Participants were asked to complete daily sleep logs and helping behavior questionnaires across 4 days, allowing for the assessment of free living, natural variations in both sleep, and helping choices across the micro-longitudinal assessment period. (**B**) Reduced sleep quality from 1 night to the next was associated with a significant decrease in helping choices from 1 corresponding day to the next and vice versa (β = 0.02 ± 0.01, *P* < 0.05, see Results for the full model). Violin plots depict the change in next-day helping behavior between the maximal and minimal sleep quality nights for each participant across the study period. *P < 0.05. Individual data presented in this figure can be found in S2 Data.

Supporting the experimental prediction, worse sleep efficiency from night to night was associated with next-day decreases in the desire to help others, and vice versa (within-person effect, *β* = 0.02 ± 0.01, *P <* 0.05, see **Fig 2** and S2 Data, all models accounting for age, sex, and survey version, R^2^ = 0.72). Reduced helping was further evident across (in addition to within) individuals, such that worse sleep efficiency overall was associated with a diminished desire to help others (between-person effect, *β* = 0.04 ± 0.01, *P <* 0.001). These effects were significant and independent of changes in sleep quantity (*P* > 0.28, see Methods and **S1 Fig**), and remained significant when accounting for trait empathy scores and daily changes in mood (within-person effect, *β* = 0.02 ± 0.01, *P* < 0.05; between-person effect, *β* = 0.03 ± 0.006, *P* < 0.001). Such findings suggest that poor sleep, either across individuals or relative to one’s own habitual sleep profile, significantly and robustly reduces prosocial helping. Distinct from prior studies that linked insufficient sleep to several different prosocial behaviors [31,40], there was no robust association between habitual sleep quantity and an individual’s desire to help others.

Whether an individual had engaged in helping others the day prior could change their proclivity to help people the following day. To test this possibility, the statistical model was adjusted to include one’s degree of helping choices the previous day. When accommodating for the extent of prior helping choices, worse sleep efficiency at night still predicted a consequential reduction in next-day helping desire (within-person effect, *β* = 0.04 ± 0.01, *P* < 0.01; between-person effect, *β* = 0.02 ± 0.004, *P* < 0.001). Therefore, an individual’s recent history of helping choices does not appear to significantly influence the impact of inadequate sleep on subsequent future helping.

Study 3 assessed the experimental prediction that the loss of 1 h of sleep opportunity decreases real-world behavioral acts of altruistic helping at a larger societal level. The prediction was tested using the manipulation of DST—a paradigm previously implemented to examine the impact of sleep loss on vehicle accidents, cardiovascular events, and aspects of mental health [41–43]. Real-world altruistic helping was quantified by assessing over 3 million charitable donations made between the years 2001 to 2016 in the United States (US) (https://www.donorschoose.org/). Analyses focused on donations during the transition to DST in the spring of each year in observing US states. To avoid the confound of donation amounts varying significantly by season (see Methods and **S2 Fig**), analyses were limited to the month before and after the DST transition each year (i.e., the 4 weeks before and after the second Sunday of March since 2007 or the first Sunday of April before that).

**Fig 3:**
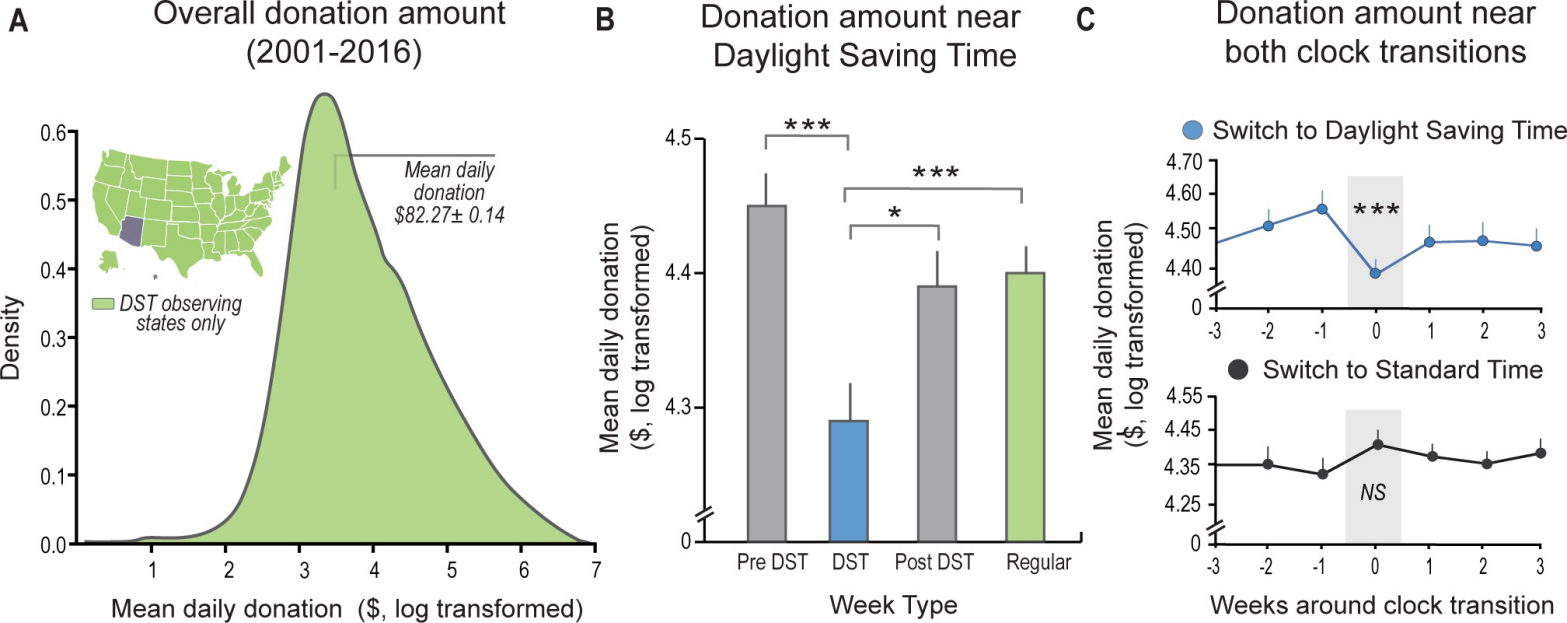
Online donation behavior—Study 3. (**A**) Overall distribution of donation amounts obtained from US states that observe DST, from 2001 to 2016. Light green inset highlights DST-observing states (i.e., excluding Arizona and Hawaii). (**B**) Donation amount was significantly lower in the week of DST transition (light blue) relevant to other weeks in the surrounding months (β_DST week_ = −0.11 ± 0.04, *P* < 0.005, adjusted for donation day, month, and year, see Methods). (**C**) The reduction in donation amount observed in the weeks around DST (top panel, centered around the third week of March) was not evident in the transition to ST (bottom panel, centered around the second week of November), suggesting that insufficient sleep triggered by the transition to DST uniquely impacts donation behavior. *P < 0.05, ***P < 0.005; error bars reflect standard error of the mean. US base layer map was plotted using the free and open-source Plotly library for python (https://plotly.com/python/maps/). Individual data presented in this figure can be found in S3 Data. DST, daylight saving time; ST, standard time; US, United States.

Fitting the experimental prediction, the transition to DST was associated with a significant decrease in the altruistic decision to give away money (make donations) compared to the weeks either before or after the transition (*β*_DST week_ = −0.11 ± 0.04, *P* < 0.005, see **Fig 3** and S3 Data, all models controlling for donation day, month, and year, see Methods). For reference, the size of the sleep effect represents approximately a 10% reduction in donation amounts.

Neither the weeks prior to nor after the DST transition showed a significant change in donation amount compared with any other week during that time (*β*_prior week_ = 0.05 ± 0.04, *P* > 0.1 and *β*_post week_ = −0.006 ± 0.04, *P* > 0.8). Therefore, idiosyncratic differences in donation amounts from one week to the next do not appear to best account for the DST-specific decrease in the human act of donation gift giving.

Three additional control analyses sought to examine nonspecific effects of time of year on donation amounts unrelated to changes in sleep opportunity. The first analysis examined donations from US states that do not observe DST (i.e., Arizona and Hawaii). This analysis determined whether there is something unique about the time period surrounding the DST transition, rather than the loss of sleep opportunity that accompanies it, that could alternatively explain the reduction in donation giving. Counter to this alternative possibility, no significant differences in donation amounts were observed during the DST transition week in states that did not experience a clock change and thus a 1-h reduction in sleep opportunity (*β* = 0.02 ± 0.08, *P* > 0.7, including the same covariates as the main model, see Methods and **S3 Fig**).

The second control analysis examined whether donation amounts varied in the weeks surrounding the transition back to standard time (ST) in the fall, a time when sleep duration is not curtailed. Of note, since the opportunity to gain an extra hour of sleep may not always be taken (in contrast to the nonnegotiable imposed loss of sleep opportunity caused by the transition to DST), the transition to ST has consistently been weaker or nonsignificant in terms of demonstrating a beneficial sleep effect [41,44]. Likewise, the week of ST transition did not significantly change mean donation amounts, relative to any other week during that time (*β* = −0.03 ± 0.04, *P* > 0.4, see Fig 3C).

The final control analysis examined whether donation amounts might be affected not by the loss of 1 h of sleep opportunity, but instead, by the loss of 1-h available opportunity to make donations (given that the day of DST transition is technically a 23-h day). When accounting for the number of donations (reflecting reduced available time to donate), the results remained as before: a significant decrease in altruistic acts of donation giving in the week of the transition to DST relative to the surrounding months (β_DST week_ = −0.09 ± 0.04, *P* < 0.05). Likewise, the transition to ST similarly remained nonsignificant as in the original findings when adding the covariate of the number of donations (β_ST week_ = −0.06 ± 0.04, *P* > 0.14).

## Discussion

Taken together, findings across all 3 studies establish insufficient sleep (both quantity and quality) as a degrading force influencing whether or not humans wish to help each other, and do indeed, choose to help each other (through real-world altruistic acts), observable at 3 different societal scales: within individuals, across individuals, and at a nationwide level.

Study 1 established not only the causal impact of sleep loss on the basic desire to help another human being, but further characterised the central underlying brain mechanism associated with this altered phenotype of diminished helping. Specifically, sleep loss significantly and selectively reduced activity throughout key nodes of the social cognition brain network [33] (see Fig 1B) normally associated with prosociality, including perspective taking of others’ mental state, their emotions, and their personal needs [16–19]. Therefore, impairment of this neural system caused by a lack of sleep represents one novel pathway explaining the associated withdrawal of helping desire and the decisional act to offer such help.

Of note, the neuroimaging task used in Study 1 focused on the prosocial skill of inferring the personal attributes of other people, a paradigm that robustly activates the social cognition network [17,45–49]. Behaviorally, this paradigm entails the central act of comprehending the mental state(s) of other individuals (itself known to be sleep sensitive [50–53]). Furthermore, this function forms the basis for inferring another person’s needs and goals, and from that, the choice to help them [54,55]. The fMRI task therefore assessed activity within the social cognition network during the act of comprehending another’s mental state, rather than targeting overt incentivized helping that could possibly bias activity linked to reward-leveraged choices [21,22,56]. Nevertheless, next-step assessments that require overt altruistic decision-making (e.g., giving money to aid others in need) will better clarify the neural impact of sleep loss on processes involving incentivized prosocial helping that include reward signaling [56].

When considering the impact of a lack of sleep on helping, it is plausible that changes in non-social factors, such as attention or effort, or changes in affective measures of mood or empathy, could also influence the desire to help others [57,58]. However, covariate analyses indicated that the impairments in helping behavior following sleep deprivation remained significant when controlling for changes in mood, effort, attention, and trait empathy. Of similar specificity, only task-related changes in activity within the social cognition brain network, and no other functional network assessed, predicted the change in helping choices, further suggesting that sleep deprivation-induced alterations within the social cognition network activity may best account for the observed changes in altruistic helping desire.

Consistent with prior reports demonstrating the detrimental impact of sleep quantity on social behaviors [31,40], Study 1 demonstrated that the total absence of sleep across a single night casually impairs the desire to help others; i.e., the presence of some period of sleep (i.e., some duration of sleep time is necessary for this prosocial feature, without which, helping behavior is significantly withdrawn). However, the findings of Study 2 add to this narrative, emphasizing that the quality of someone’s sleep (here, sleep efficiency) is similarly and robustly associated with the next-day withdrawal of helping. Therefore, the findings of Study 2 suggest that once sleep duration rises above some basic nominal amount (accomplished in Study 2, but prevented in Study 1), then the quality of that sleep most accurately predicts the profile of altruistic desire the next day, a finding corresponding to other impairment in socioemotional functions linked to poor sleep quality [28–30].

Studies 1 and 2 further demonstrate that the effect of sleep on prosocial helping is bidirectional. Specifically, not only did deficiencies in sleep reduce human helping, but when adequate sleep was (re)established, the desire to help others was reestablished. This directional effect was also evident in Study 3, wherein the transition to DST impaired donation amount, though the effects following the return to ST did not reach statistical significance. A plausible reason for the stronger effect of DST relative to ST (which has been a common feature in numerous studies assessing functions different to helping [41,43,59,60]) is that the option to sleep an extra hour following the transition to ST is not always taken, in contrast to the imposed loss of sleep opportunity caused by the transition to DST. Alternatively, it is known that individuals suffer less sleep disruption following a phase delay (which would be more similar to the phase shift associated with the switch to ST), relative to a phase advance (more akin to the DST transition challenge) [61,62]. Such a difference in adaptation may also contribute as an added factor explaining the greater effect size observed in altruistic helping following the DST transition relative to the ST transition.

Study 3 complemented and extended Studies 1 and 2 by demonstrating that a 1-h reduction in sleep opportunity is associated not just with a reduced desire to help others, but an impairment in the decision to help other individuals in need by way of monetary donations. Specifically, the impact of sleep loss manifests in the real-world abatement of altruistic helping, evidenced through a reduction in the consequential act of philanthropic giving on a nationwide scale (US). Similar to the causal experimental manipulation of sleep in Study 1, Study 3 therefore offered a causal assessment of how a lack of sleep opportunity altered the subsequent act of helping (the altruistic giving away of money). Perhaps most critical, this effect on real-world consequential behavior was not through the extreme and less common experience of total sleep deprivation, but instead, the societally pervasive loss of just 1 h of sleep, and for 1 night. Indeed, the shift to DST has consistently been demonstrated to involve a 40 to 60 min reduction in total sleep amount, coupled with a 10% reduction in sleep efficiency due to increased sleep fragmentation [59,60,63,64].

Findings from Study 3 additionally establish that the withdrawal of helping associated with sleep loss does not depend on direct personal interaction with those in need of help, since such donation gifts of money were absent of interpersonal or in-person contact. As such, the rescinding of help associated with insufficient sleep is not reasonably explained by under-slept individuals simply wishing to be alone and therefore excising themselves from social contact, making them physically unavailable to help. Instead, data from Study 3 suggests a broader and more intrinsically determined phenotype of impaired socioemotional functioning, one that is not reliant on actual social interaction.

Mechanistically, emerging evidence indicates a role of acute stress, including increased cortisol release, in reducing prosocial behavior [65], charitable giving [66,67], and performance in Theory of Mind tasks [68], while increasing egoistic choices in moral dilemmas as levels of cortisol rise [69]. Such findings are relevant since sleep loss increases autonomic physiological arousal related to greater sympathetic nervous system dominance [29] as well as increases hypothalamic-adrenal stress axis (HPA) activation leading to higher cortisol levels [70]. Therefore, increased sympathetic autonomic dominance, hyperactivation of the HPA axis, and associated increase in cortisol may be one peripheral body pathway through which a lack of sleep impairs the central brain-determined choices of prosocial human helping, shifting individuals into a more egoistic (rather than benevolent, altruistic) state of action repertoires.

Related, insufficient sleep impairs mood. Furthermore, impaired positive mood can influence helping, in part by lowering empathic sensitivity to the needs or distress of others [71], also seen in states of clinical depression [72–74]. Though the reductions in helping behavior observed in Studies 1 and 2 remained significant when accounting for corresponding changes in positive and negative mood, affective changes linked to insufficient sleep may nevertheless play a moderating or mediating role in the broader deficits reported in varied prosocial behaviors linked to a lack of sleep, beyond the abatement of helping.

The multivarious associations between sleep loss and diminished helping behavior across Studies 1–3 offer next-step testable hypotheses, perhaps most importantly those linked to downstream consequences. For example, helping behavior is a foundational element of modern societal fabric, including numerous acts of human civility [3,75]. Early evidence already suggests a potential link between sleep and the degradation of this core foundational element, wherein sleep loss predicts a reduction in voter turnout during national elections (e.g., US, German) [40]. Aligning with the findings of Studies 1–3, such evidence helps establish the broad spectrum of impaired prosocial behaviors of varied forms that are linked to an equally varied range of sleep deficiency, from total sleep deprivation [32], partial and modest sleep restriction [31], as well as insufficient sleep caused by circadian disruption [31,40].

Another relevant issue concerns the decline in giving amounts (relative to income) over the last 60 years. This trend has been attributed to shifts in societal norms and economic structures [75]. Data from all 3 studies raise the possibility that corresponding reductions in sleep, which have occurred in temporal lockstep over this same timeframe [76], may be an additional and previously unconsidered contributing factor in the downward trend in philanthropic giving and thus in the decline of the “helping economy.”

More generally, our findings suggest a model in which sleep, when present in sufficient quantity and quality, can preserve and enhance the macrosocial force that is helping, and when sleep becomes deficient in amount and quality, imposes an impediment to this prosocial, societal necessity. Interestingly, communities suffering from worse sleep quantity and quality express lower overall levels of social capital [77–79]—a measure of the resources available to individuals through help-based social networks [80]. Considering that more than 50% of individuals across numerous first-world nations report not getting sufficient sleep during workdays (National Sleep Foundation, 2013), this proposition may warrant greater investigation at a societal level. If found to be true, it may necessitate methods to enhance sleep awareness and the development of policies that improve sleep opportunities for individuals within affected communities [81].

While our findings establish sleep loss as a previously unrecognized factor influencing whether humans offer or withdraw help at numerous levels of civilized interaction, they conversely highlight adequate sleep as a modifiable factor to promote greater helping. This is in contrast to more fixed features, such as personality traits or broader cultural edicts that are likely to be challenging to target as interventional methods for promoting prosocial behavior. Therefore, interventions and/or policies that aid individuals, communities, and societies to obtain sufficient sleep may lead humans to help one another with greater alacrity and consistency, fitting the original assertion listed at the start of this manuscript by Muhammad Ali.

## Materials and methods

### In-laboratory experiment (Study 1)

#### Participants

Twenty-four healthy adults, ages 18 to 26 years (mean ± SE: 20.6 ± 0.35 yr, 54% female) completed a counterbalanced, randomized crossover design (described below). Participants abstained from any psychoactive drugs (including caffeine and alcohol) for 24 h before each study session. Participants’ habitual sleep–wake rhythm was monitored for 3 nights prior to study participation verified by sleep logs and actigraphy (a wristwatch movement sensor, sensitive to wake and sleep states). Exclusion criteria, assessed using a prescreening questionnaire, included a history of sleep disorders, neurologic disorders, closed-head injury, Axis 1 psychiatric disorders, history of drug abuse, and current use of antidepressant or hypnotic medication. Participants were also excluded from entering the study if they reported: sleeping less than 7 h per night, traveling across time zones in the past month, doing shift work in the past year, having their bedtime and/or wake time change by more than 2 h more than 3 times a week or consuming 3 or more daily caffeine-containing drinks. The study was approved by the local human studies committee of the University of California Berkeley, with all participants providing written informed consent.

#### Experimental design

Following successful completion of screening, participants entered a randomized crossover study design involving two sessions, conducted in a counterbalanced order: one after a rested night of sleep and one after 24 h of sleep deprivation. Participants were randomly assigned to start with either a sleep-deprived or a sleep-rested session followed by the crossover session, separated by at least 7 days. Upon entering the study, and prior to any sleep manipulation, participants completed the Interpersonal Reactivity Index (IRI [82]) in order to measure interindividual differences in empathy.

Within each session, helping behavior was assessed in the morning (between 9 to 11 AM), using the helping behavior questionnaire (details below). In addition, mood states were measured twice in each session using the Positive and Negative Affect Schedule (PANAS [83]), first in the evening prior to any sleep manipulation (between 8 to 10 PM) and again in the morning following both sleep sessions (between 8 to 9 AM).

In the sleep-deprived session, participants arrived at the laboratory at 9:30 PM and were continuously monitored throughout the enforced waking period by trained personnel. During the sleep deprivation period, participants engaged in a limited set of activities such as studying, being online, reading, or watching movies. The following morning at approximately 10:00 AM (±45 min), participants performed a social judgment fMRI paradigm inside the scanner (details below). In the sleep-rested session, participants arrived at the lab at 7:00 PM and were prepared for an ambulatory electroencephalography (EEG) polysomnography recording, after which they were sent home to sleep allowing for more naturalistic measurement. The next morning, participants returned to the laboratory and had the electrodes removed. Participants then performed the same activities as those described above in the sleep deprivation condition, starting at the same matched circadian time.

#### Helping behavior assessment

Helping behavior was assessed using a 40-item questionnaire sourced from the Self-Report Altruism Scale [84], a scale that is also part of the Prosocial Personality Battery [85,86]. Both are frequently used in studies assessing prosocial and altruistic helping behaviors [71,86–91]. Each item described a social situation requiring various types of help (e.g., “If I was in a hurry to get to work and someone stopped me to ask for directions I would…” or “I would help a stranger struggling with her grocery bags to carry them,” for a full list of the items see Table B in S1 Text). For each statement, participants were asked to indicate how they would respond to the social situation at this moment in time using a 5-box vertical scale ranging from “I would definitely help” or “I would stop to help” (wording was tailored to each scenario, see Table B in S1 Text) to “I would not help” or “I would ignore them.” The reliability of this scale in this sample was high (Cronbach’s alpha = 0.82 in the sleep-rested session and 0.87 in the sleep-deprived session).

The helping requests in the assessment were equally divided between strangers and familiar others to control for the known impact of familiarity on helping behavior [2] (see Note A in S1 Text for an analysis of familiarity effects). For example, “I would offer my seat on a crowded bus to a 60-year-old woman” reflects a social scenario involving a stranger while “If a coworker who lives near me asked me to give him/her a ride home I would…” involves a familiar other.

In addition to these social situations, 10 nonsocial control items that targeted factual questions were included in the questionnaire (e.g., “Is Denmark larger than Sweden?”), requiring a yes/no binary reply. Social items only were subsequently reversed scored (such that higher scores denote greater helping, range 1 to 5) and averaged for each participant. One participant was excluded from the analysis due to partial completion of the questionnaire (35% missing values). The helping behavior questionnaire included 2 separate balanced versions (each with different 30 social and nonsocial statements), such that participants never replied to the same questionnaire twice. Versions were counterbalanced across participants and sessions. Notably, no significant order effects were found when comparing the participants who completed their sleep deprivation session first, relative to those who completed it second (mean difference = −0.05, 95% CI = [−0.68, 0.58], t = −0.18, *P* = 0.9) and similarly for the order of the sleep-rested session (mean difference = −0.32, 95% CI = [−0.82, 0.17], t = −1.37, *P* = 0.2).

#### Effortful behavior control task

Sleep loss can impact the willingness to exert effort [57] and could therefore indirectly influence the desire to help others. To explore this factor of effort, participants in Study 1 performed an incentivized effort task in both the sleep-rested and sleep deprivation sessions (similar to a grip effort task [92]). During each session, participants were required to use their right index finger to press the “m” key at variable speeds for a certain amount of timed effort intervals.

The effort task involved 3 blocks. The first block lasted 3 min and asked participants to press the “m” key at a speed faster than 4 presses per second. The second block required participants to choose between hard trials (4 to 6 key presses per second) or easy trials (2 presses per second) for a total of 20 trials. Here, participants were offered monetary rewards based on their choice: 10 cents for an easy trial and 10 to 80 cents for hard trials. In the third and final block, participants were asked to press “m” at a constant speed of 4 strokes or faster per second for as long as they wished, up to a maximum of 8 min. Each minute rewarded participants with $1 for their effort, such that the maximum reward available was $8. Participants were informed they could stop at any time during this last block and would be rewarded based on the time they had already spent. This “time on task” variable indicated the level of sustained effort participants were voluntarily willing to exert and was therefore used to assess participants’ volitional effort levels, subsequently factored into the main analyses examining helping. Results of the time on task analysis indicated that participants chose to exert less effort following sleep deprivation (time on task duration in minutes (means ± SE: SR = 5.82 ± 0.68, SD = 4.03 ± 0.62, a 31% reduction; *P* = 0.015, d = −0.57), yet as described in the Results, differences in helping behavior remained significant after statistically accounting for changes in effort.

#### fMRI social judgment paradigm

The relationship between neural activity within the social cognition network and prosocial behavior was examined using a well-documented mentalizing task, wherein participants were asked to think about personal attributes of social targets [17,45–49]. This allowed for an ecological assessment of social cognition brain activity without any overt biases of knowing the study’s primary motivation which was that of helping choices. Such a design offers an orthogonal approach to examining the neural correlates of prosocial behavior triggered by empathy [20,21] or incentivized donation choices [22,23], by targeting a key foundational process of prosocial behavior: the comprehension and understanding of another’s mind [37,39].

During fMRI scanning, participants viewed 48 experimentally controlled information cards depicting various adults based in the US (including name and profession, represented by a silhouette black and white image to avoid visual face biases, see example in **S4 Fig**). Participants were asked to make explicit personality trait judgments for each individual presented in each trial, a process that evokes a robust engagement of the social cognition brain network [46,47,49]. For each information card trial, participants made social assessment ratings regarding the level of perceived competence or warmth of each individual based on the details provided to the participant on that trial (ranging from 0 to 6, warmth and competence ratings randomized across runs). There were no significant differences between the sleep deprivation and sleep-rested conditions for scores of either competence (SR = 3.28 ± 0.09, SD = 3.27 ± 0.09, *P* = 0.9) or warmth (SR = 2.95 ± 0.11, SD = 2.84 ± 0.08, *P* = 0.6) ratings.

In each trial, the information card was presented for 3 s, followed by the social judgment response screen for 4 s and an inter-trial fixation period (jittered, 2 to 6 s for optimal event-related fMRI design). For the nonsocial control trials, a total of 32 additional information cards of equivalent size were used that depicted various objects using a black and white image (e.g., vacuum cleaner, guitar). In these object trials, participants were asked to rate how old they believed the objects to be, using a similar scale of 0 (very old) to 6 (very new). As with previous such fMRI paradigms [93,94], these nonsocial cards provided an on-task comparison with the human information cards (i.e., human > object judgment), allowing for the discrimination of brain activity that is unique to the processing of social stimuli above and beyond the presentation of a visual object or other nonspecific task demands.

The in-scanner social judgment paradigm contained 2 repeatable versions, each including a different set of 80 information cards (48 humans and 32 objects, though see Note B in S1 Text for a control fMRI analysis in which human and object trials are equally balanced). The version used was counterbalanced across participants, such that each version was viewed in a sleep-rested session for half of the participants and in a sleep-deprived session for the others. In each session, the information cards were presented in 2 runs, with human and object cards presented in a randomized order within each run. The start of each run contained a 10-s fixation block, allowing for a steady-state equilibrium of the BOLD fMRI sequence. Stimulus presentation and response collection were controlled by PsychoPy [95].

Similar to prior studies, helping behavior was assessed outside the scanner [20,21] (see Helping behavior assessment above for full details) to enable the use of real and multidimensional scenarios of helping others [6], rather than focusing on a predetermined domain of prosocial behavior (e.g., providing financial support [22,56]). This assessment further provided an ecological measure of helping choices, allowing for time-dependent processes (e.g., personal values, attitudes, dispositions) to come into play [96], unconstrained by typical event-related fMRI limitations.

#### fMRI acquisition and analysis

Blood oxygenation level-dependent contrast functional images were acquired with echo-planar T2*-weighted (EPI) imaging using a Siemens 3 Tesla MRI scanner with a 12-channel head coil. Each image volume consisted of 37 descending 3.5 mm slices (96 × 96 matrix; TR = 2,000 ms; TE = 22 ms; voxel size 3.5 × 3.5 × 3.2 mm, flip angle = 50°, 0.3 mm interslice gap). One high-resolution, T1-weighted structural scan was acquired at the end of the sleep-rested session (256 × 256 matrix, TR = 1,900; TE = 2.52; flip angle = 9°; FOV 256 mm; 1 × 1 × 1 mm voxels).

Preprocessing and data analysis were performed using fMRIprep v1.25 [97] and Statistical Parametric Mapping software implemented in Matlab (SPM12; Wellcome Department of Cognitive Neurology, London, UK). Using fMRIprep, T1-weighted (T1w) images were corrected for intensity non-uniformity, skull-stripped, and spatially normalized to the ICBM 152 Nonlinear Asymmetrical template. Brain tissue segmentation of cerebrospinal fluid (CSF), white matter, and gray matter were then performed on the brain-extracted T1w. Functional image preprocessing included coregistration with the T1w structural scan, slice-time correction, resampling to MNI152NLin2009cAsym standard space (voxel size 2 × 2 × 2 mm), and spatial smoothing (6-mm Gaussian kernel), using the fMRIprep pipeline. To control for head motion and physiological artifacts, 13 nuisance regressors were calculated as well, including 6 rotation and translation parameters, framewise displacement (FD), and the first 6 principal components of the anatomical CompCor pipeline (taking into account CSF and white matter signals).

Following preprocessing, a standard general linear model (GLM) was specified using SPM for each participant to investigate the effects of interest. Contrasts were created at the first level focusing on human < > object judgments to elucidate regions sensitive to social cognition. The resulting contrasts were then taken through to a second level, random-effects analysis to assess group-level effects, examined using a paired *t* test (Sleep Rested < > Sleep Deprived). Analyses focused a priori on activity in a set of brain regions comprising the social cognition network, regions that have been implicated in studies of social cognition and helping behavior [20–23]. These regions include: bilateral TPJ [MNI coordinates: −48; −60; 32 left, 56; −60; 22 right], dorsomedial PFC [2; 56; 22], ventromedial PFC [2; 44; −18], precuneus [2; −52; 32], bilateral middle temporal sulcus [−52; 2; −26 left, 56; −2; −24 right], and right inferior frontal gyrus [52; 30; −8].

Regions of interest (ROIs) were derived using the NeuroSynth framework [98], a large-scale automated meta-analysis tool for neuroimaging data. An activation map was calculated from 80 social cognition studies that were corrected for multiple comparisons (FDR < 0.01). A minimal size of 150 voxels per cluster was further applied to the activation map, resulting in a mask of 8 binarized ROIs (see Table C in S1 Text for a complete list of ROIs). Analyses focused on averaged activity across all ROIs in order to examine task-evoked network-level activation and avoid multiple comparisons across separate ROIs [99]. Using the binarized mask, condition-specific (human > object) activity was extracted and then compared between sleep conditions. Beyond these network-level ROIs, non-a priori whole-brain results are provided in Table D in S1 Text for results-reporting completeness but are not discussed further.

Finally, to examine the issue of specificity, two post hoc analyses were conducted. The first explored activity within six additional standard brain networks using the same analysis steps described above. The networks were derived from a validated seven-network parcellation [100] and included the limbic, frontoparietal, ventral/dorsal attention, somatomotor, and visual networks (absent the default mode network, given its substantial overlap with regions of the social cognition network [101,102]). None of the activation patterns in these networks showed a significant main effect of sleep deprivation during the social judgment task, and similarly, sleep loss changes in activity within each of these networks did not predict the degree of impairment in helping behavior (all *P* > 0.1, corrected for multiple comparisons, see Table A in S1 Text).

The second analysis examined the possible influence of attention. Reaction time is often used as a proxy for attentiveness and attention [103–106] and is commonly included as a cofactor variable in typical fMRI analyses to control for attention [107–109]. Using this same approach, single-trial reaction times during the fMRI scanning task were extracted per individual and per session (sleep rested, sleep deprived). These reaction times were then included as a “nuisance regressor” in the single-subject level of the fMRI analysis, further controlling for measures of movement and physiological confounds as in the main analysis. When controlling for trial-specific reaction times, the results of the main findings remained similarly significant, evincing a significant and selective reduction in social cognition network activity following sleep deprivation, relative to the sleep-rested condition (mean change = −0.46 ± 0.17, *P* = 0.02, d = 0.7). Moreover, activity in the social cognition brain network remained significantly associated with helping behavior when reaction times were added as a cofactor in the analysis model (R = 0.43, *P* = 0.04).

#### Sleep recordings

Sleep was recorded using standard polysomnography including EEG, electromyography (EMG), and electrooculography (EOG) recordings. EEG was recorded from 11 scalp electrodes (Fp1, Fp2, F3, F4, F7, F8, C3, C4, P3, P4, and Oz; International 10–20 System), referenced to left and right mastoid (A1, A2). EEG signals were sampled at 200 Hz. Polysomnographic recordings were scored according to standard criteria [110]. Sleep statistics are provided in Table E in S1 Text and conform to population norms for this age range [111].

### Online micro-longitudinal study (Study 2)

#### Participants

Study 2 tested whether more modest night-to-night variability in self-reported sleep efficiency and sleep duration predicted day-to-day changes in helping behavior the next day. Unlike the experimental sleep manipulation of Study 1, Study 2 examined sleep variations under free-living conditions. A total of 171 participants (age 36.96 ± 0.73 yr, 41.2% female) signed up for this 4-day study using Amazon Mechanical Turk (MTurk)—a platform where individuals can perform online tasks for a specified reimbursement (here, $4.25 to 5.75 depending on the final number of daily surveys). Enrollment was restricted to those with IP addresses in the US and a prior online MTurk approval rating of 95% or higher. Additional exclusion criteria included a current diagnosis of an Axis 1 psychiatric disorder and/or the confirmed diagnosis of a sleep disorder. Participants were also excluded from further analysis if they completed only 1 daily survey, which would otherwise have prevented sufficient variability in assessing within-person effects (and see the robustness assessment regarding the main regression using a minimum of 3 or 4 nights of data in Note C in S1 Text). Furthermore, participants whose sleep logs reported extreme sleep duration values were similarly excluded (less than 3 h or more than 12 h). The final sample, therefore, included 136 participants (mean age ± SE = 37.83 ± 0.87 yr, 41.9% female), yielding a total of 441 valid observations across the study period.

#### Study design

Following recruitment, participants were asked to complete validated daily sleep diaries (see Table F in S1 Text), quantifying their sleep across four consecutive nights. The next day, participants completed an assessment of helping behavior using a shorter form of the helping questionnaire applied in Study 1 and described above. The short-form version included 10 social items depicting requests for help, presented in random order, and counterbalanced for familiarity such that requests from strangers and familiar others were equally represented in each daily survey. Each survey day included a different version of the short-form questionnaire depicting different social scenarios. Similar to Study 1, reliability measures for this scale were also strong (Cronbach’s alpha = 0.87 for version 1, 0.85 for version 2, 0.88 for version 3, and 0.9 for version 4). The longitudinal nature of Study 2 further allowed for an examination of test-retest reliability in this sample, which was 0.79 for the first consecutive days of the survey, 0.78 for days 2 to 3, and 0.72 for days 3 to 4.

To measure helping behavior with respect to prior sleep, the survey was only available online during a specific time window in the morning (until 1 PM local time), and participants were requested to complete the survey as close as possible to their wake-up time. In addition to the key outcome variable of helping behavior, measures of mood were also collected in each daily survey using the short PANAS questionnaire [112] described above. Finally, trait empathy was assessed upon entry to the study using the IRI [82], as in Study 1.

#### Data preprocessing and analysis

Analyses focused a priori on sleep efficiency and sleep duration, given previous work linking both sleep parameters to social and interpersonal functioning [28,30]. Sleep efficiency was measured using participants’ daily sleep diaries, based on the percent of time asleep out of total time in bed (i.e., total time in bed minus sleep latency and time spent awake after sleep onset, mean ± SD = 90.02 ± 9.36%). Sleep duration was calculated as the total time elapsed from sleep onset to wake time minus sleep latency and time spent awake after sleep onset (mean ± SD = 429 ± 68.8 min).

A linear mixed-effects model was calculated to test whether night-to-night variability in sleep efficiency and sleep duration, within participants, predicted day-to-day changes in helping behavior the next day. The predictors of sleep efficiency and duration were calculated for both between- and within-person effects using person mean centering [113] (see Statistical analyses below and **S1 Fig**). The between-person effect refers to a person’s average over the study period (e.g., mean sleep duration/efficiency across 4 days), while the within-person effect refers to that person’s deviation from their average on a particular day (mean deviation in sleep efficiency = ± 2.03%, mean deviation in sleep duration = ±35.48 min, or 8.2% of total sleep time). All assessment days were weekdays to avoid concerns of weekend changes in sleep patterns. All linear mixed-effects models were adjusted for age, sex, and survey version.

While the main model focused on sleep duration and efficiency, it is important to note that circadian rhythms and circadian disruption also influence emotional and mood states [114–117]. We therefore, sought to further explore the contribution of circadian influence to helping behavior. For each participant, the measure of mid-sleep was calculated as the halfway point between sleep onset and sleep offset time during the study period, a marker of habitual circadian phase [118,119]. Similar to the main analysis, mid-sleep was assessed for both between- and within-person effects. Adding these parameters to the main model, there was no significant effect of circadian phase (mid-sleep) on helping behavior (within-person effect, β = −0.04 ± 0.03; between-person effect, β = 0.03 ± 0.04, both *P* > 0.25).

### Online donations database (Study 3)

#### Donation data and analysis

Study 3 tested the prediction that the loss of 1 h of sleep opportunity (due to DST) resulted in a real-world, large-scale decrease in helping behavior. Data were obtained from an online database of donations made between the years 2001 to 2016 in the US, via the DonorsChoose website, a platform that helps raise funds for school projects in the US (e.g., buy books, get supplies for a science project).

A total of 6,211,956 donations were available for analysis, including information about donor location, the timestamp of each donation, and the project each donation was intended to fund. Donations were excluded from further analyses if they did not include information on date/time or on donor location or were intended for projects that were not eventually funded (e.g., projects that expired before meeting their funding goal or were still not funded at the moment of download). Donations for projects that lasted less than a day were also excluded to allow for more stable predictors of donation behavior over time (including possible effects of sleep).

The main model focused on nationwide donations coming from states that observe DST (i.e., excluding Hawaii and Arizona), totaling 3,871,500 eligible donations (average daily donation amount $82.27 ± 0.14). For each donation, the following information was calculated and used in the statistical analyses: the day of the week/month/year of the donation and the time of day the donation was made. In accordance with prior reports [41,43,63,120,121], analysis focused on the weekdays following the transition, as both the ST and DST transitions result in sleep consequences lasting up to 5 days before sleep onset and offset times revert, and habitual sleep patterns return [59,122]. Analyses therefore focused on a robust window that spanned multiple days of assessment (see Note D in S1 Text for a secondary analysis focusing on post-transition Monday alone).

Using timestamp data of each donation, analyses tested the hypothesis that during the week of the transition to DST (the weekdays following the second Sunday of March since 2007 or the first Sunday of April before that), the corresponding loss of 1 h of sleep opportunity would significantly decrease altruistic helping behavior reflected in lower donation amounts. The statistical models examined the 4 weeks before and after the DST transition to avoid annual seasonal effects on donation amounts (e.g., donation amounts are lowest during the summer vacation when school is out, see **S2 Fig**). Donations were then aggregated to a daily average amount across the examined period (2001 to 2016, total number of days = 18,454) and subsequently log-transformed before being implemented in a multiple regression model (see Statistical analysis below). Donation data were filtered to exclude extreme outliers (above or below 3 standard deviations from the mean), most of which from a small number of donors giving very large amounts of money (e.g., more than $100,000 in a single donation) or from days that included extremely high numbers of recorded donations. Following these criteria, a total of 3,420,996 donations were aggregated across 18,034 days and utilized in the analyses.

#### Time of year control analysis

In order to test for nonspecific effects of time of year on donation amounts, 3 additional models were constructed. The first model examined whether the mere switching of the clock might impact donation behavior irrespective of sleep changes by focusing on the months surrounding the transition back to ST (the weekdays following the first Sunday of November since 2007 or the last one in October before that). Analyses for this model similarly focused on the 4 weeks before and after the transition to ST as the key predictor of interest and controlling for the same covariates as the main model.

The second model examined possible time of year effects on donation behavior, probing whether the months of March/April when DST transition takes place, might impact donation behavior irrespective of changes in sleep. This model focused on the same timeframe as the main model (i.e., the weekdays following the second Sunday of March since 2007 or the first Sunday of April before that), but was now applied to donations coming from Arizona and Hawaii, states that do not observe DST and therefore are unlikely to experience sleep loss during the transition week. Data from Arizona and Hawaii included 76,276 donations made between the years 2001 to 2016 (average donation amount $70.23 ± 1.11).

Finally, the last model accounted for time availability effects. Since the day of DST transition is technically a 23-h day, changes in the available time to make donations, and thus a reduced number of donations, could impact donation amounts irrespective of changes in sleep. To account for such effects, an additional model was analyzed that controls for the number of daily donations made during the study period, given that the act of donation gifting is indeed subject to time availability irrespective of donation amount. Analyses for this model similarly focused on the 4 weeks before and after the transition to DST/ST as the key predictor of interest, controlling for the same covariates as the main model.

### Helping assessments (Studies 1 to 3)

#### Helping behavior

The key outcome measures of helping behavior used in Studies 1 to 3 were each designed to offer different but complementary measures of helping, thus allowing us to demonstrate a broader phenotype of prosocial behavior across all studies. The outcome measures taken in Studies 1 and 2 assessed participants’ desire to help, using the helping behavior questionnaire, assessing numerous forms of helping deeds and acts common in everyday social life [6,71]. Adding to this, Study 3 examined consequential helping, measuring real-world donation behavior using an online donation database and thus reflect a decisive action that directly resulted in the financial giving of help: an altruistic one, since it importantly did not result in (nor depend on) any reciprocal direct financial gain to the donor (a core construct of altruism) [123,124]. As such, and fitting prior studies [20,21,125,126], the assessment of prosocial helping did not incentivize the choice to help others, since acts of altruism typically do not involve reciprocal material exchanges, but instead, are benevolent and unidirectional [123,124].

Thus, Studies 1 and 2 measure the motivational desire to act altruistically, with the study design choice of a helping behavior questionnaire being motivated by the need to assess a wide selection of prosocial deeds and acts common in everyday life. Broadening the aperture of helping assessment, Study 3 evaluated an altruistic, consequential helping choice, which was the decision to give away money without any incentivized reciprocal benefit.

### Statistical analyses

#### Behavioral results

To test the hypothesis of reduced helping behavior following sleep loss in Study 1, a repeated measure ANOVA was calculated, taking into account familiarity (stranger versus familiar other) across the different sleep conditions (sleep rested, sleep deprived). In case of significance, post hoc tests were computed using paired 2-sided *t* tests corrected for multiple comparisons using the Bonferroni correction. To control for changes in mood and effort following sleep loss, a multiple linear regression was applied using the difference scores of each variable (sleep deprivation, sleep rested). In this model, the intercept coefficient reflected the sleep loss effect on helping (i.e., zero denotes no effect), while the mood and effort variables were added as covariates. Associations between social cognition network activity and helping behavior were tested using Pearson’s correlation, with mean activity from the entire a priori network of interest (i.e., not only limited to the activated cluster) to avoid spurious fMRI-behavior correlations. All statistical analyses were conducted using JASP (JASP Team 2021) and the pingouin library implemented in Python [127].

#### Multilevel modeling

Linear mixed-effects models were used to determine associations between sleep and helping behavior across days in Study 2. All multilevel models were adjusted for age, sex, and survey day, with subject identifier defined as a random effect. A person-mean centering was applied to the predictor variables in all models to disaggregate the between-person and within-person effects [113]. The time-invariant person average and time-varying deviation from an individual’s average were then both included as fixed effects in the multilevel model (between- and within-person components, respectively). To control for outliers in the 2 key predictors of interest, sleep efficiency and sleep duration values that were ±3 STD from the mean were filtered prior to the final analysis. In addition to the main model, 2 additional models were calculated controlling for changes in (i) mood states (within-person change across days) and trait empathy (between person factor); and (ii) prior helping behavior (a daily variable). These models were identical to the main model in terms of the key predictors and covariates. All multilevel analyses were performed in R [128] using the “lme4,” “lmerTest,” and “sjPlot” packages [129–131]. Goodness-of-fit was evaluated using the conditional R^*2*^ [132].

#### Multiple regression

Study 3 implemented a multiple regression model to evaluate the impact of DST transition on donation behavior. The log-transformed daily donation amount in US dollars was set as the outcome variable, while the key predictor of interest was “week type” (with levels Monday to Friday before DST, DST weekdays, weekdays after DST, and any other day of the year). All models were adjusted for additional predictors influencing donation likelihood including: time of day (using 4 equal 6-h bins: morning: 6 AM to 11:59 AM, afternoon: noon to 5:59 PM, evening: 6 PM to 11:59 PM, and night: midnight to 5:59 AM), day of the week, month, year, weekend day (Saturday or Sunday), and holidays (as a binary True/False variable). For the control analysis of available time to donate, the predictor of number of donations (log-transformed) was also added to the main model. If DST transition lowered donation amount in the 5 days after switching to DST, a significant coefficient for the level “DST week” was expected compared to all other Monday to Friday in the variable “week type.” Similarly, a nonsignificant coefficient for “week type” was expected in the 2 control models exploring the transition back to ST as the key predictor or when focusing on donations from states that do not observe DST (i.e., Arizona and Hawaii). All tests of statistical significance were two-sided, and *p* values less than 0.05 were considered statistically significant. All analyses were performed in R [128] using the “lme4” and “lmerTest” packages.

## Supporting information

S1 Fig Model estimates of sleep efficiency and duration predicting helping behavior (Study 2). (**A**) Helping behavior was higher following nights of better sleep quality (within-person effect, β = 0.02 ± 0.01, *P* < 0.05) as well as in individuals who sleep better overall (between-person effect, β = 0.04 ± 0.01, *P* < 0.001). (**B**) No significant effects on helping behavior were found for sleep duration for either nightly changes (within-person effect, β = −0.0002 ± 0.0005, *P* > 0.6) or changes in sleep duration across participants (between-person effect, β = −0.0007 ± 0.0007, *P* > 0.3). Model estimates derived from an analysis of 441 observations obtained from 136 individuals across 4 consecutive days. ***P < 0.001. Individual data presented in this figure can be found in S2 Data.
(TIF)

S2 Fig Seasonality effects in donation amount (Study 3). Donation amount was highest in winter and lowest in summer (main effect of season, F (3,67) = 45.17, *P* < 0.001). Model estimates derived from an analysis of all donations obtained from US states that observe Daylight Saving Time (i.e., excluding Arizona and Hawaii). ***P < 0.001, post hoc *t* tests corrected for multiple comparisons, error bars denote standard error of the mean. Individual data presented in this figure can be found in S3 Data.
(TIF)

S3 Fig Donation amount in non-DST states (Study 3). Donation amount was not significantly different in the months surrounding the DST transition in stated that do not experience an actual clock change (*β* = 0.02 ± 0.08, *P* > 0.7, including the same covariates as the main model, see Methods). Model estimates derived from an analysis of all donations obtained from US states that do not observe Daylight Saving Time (i.e., Arizona and Hawaii). Error bars denote standard error of the mean. US base layer map was plotted using the free and open-source Plotly library for python (https://plotly.com/python/maps/). Individual data presented in this figure can be found in S3 Data.
(TIF)

S4 Fig Social judgment stimuli (Study 1). An example of the stimuli used in the social cognition task during fMRI scanning. Each stimulus depicted an individual that participants were asked to assess, including their name, professional details, and a silhouette grayscale image.
(TIF)

S1 Text Supplementary tables and notes for Studies 1–3. (DOCX)

S1 Data Excel spreadsheet containing, in separate sheets, the values for each individual for Fig 1A–1C. (XLSX)

S2 Data Excel spreadsheet containing, in separate sheets, the values for each survey observation in Figs 2B and S1. (XLSX)

S3 Data Excel spreadsheet containing, in separate sheets, the values for each donation for Figs 3B, 3C, S2, and S3. (XLSX)

## Abbreviations

CSF: cerebrospinal fluid
DST: daylight saving time
EEG: electroencephalography
EMG: electromyography
EOG: electrooculography
FD: framewise displacement
GLM: general linear model
HPA: hypothalamic-adrenal axis
IRI: Interpersonal Reactivity Index
mPFC: medial prefrontal cortex
PANAS: Positive and Negative Affect Schedule
ROI: region of interest
ST: standard time
TPJ: temporal-parietal junction
US: United States
