# Correction: Sleep loss leads to the withdrawal of human helping across individuals, groups, and large-scale societies

**DOI:** 10.1371/journal.pbio.3002394

**Published:** 2023-11-15

**Authors:** Eti Ben Simon, Raphael Vallat, Aubrey Rossi, Matthew P. Walker

The authors have updated their Data Availability statement to extend the availability of relevant code. Please see the updated statement here.

Donation data are available from the DonorsChoose website upon request (https://help.donorschoose.org/hc/en-us/articles/360047050214-Information-for-academic-researchers-data-scientists-and-developers). For Study 1 and 2 all relevant individual data and summary statistics are within the paper and its Supporting Information. Detailed analysis scripts and study material are further freely available on the Open Science Framework (https://osf.io/2973t/).

Fig 3 has been updated to fix axis scaling in parts A and B. Please see the updated Fig 3 here.

**Fig 3:**
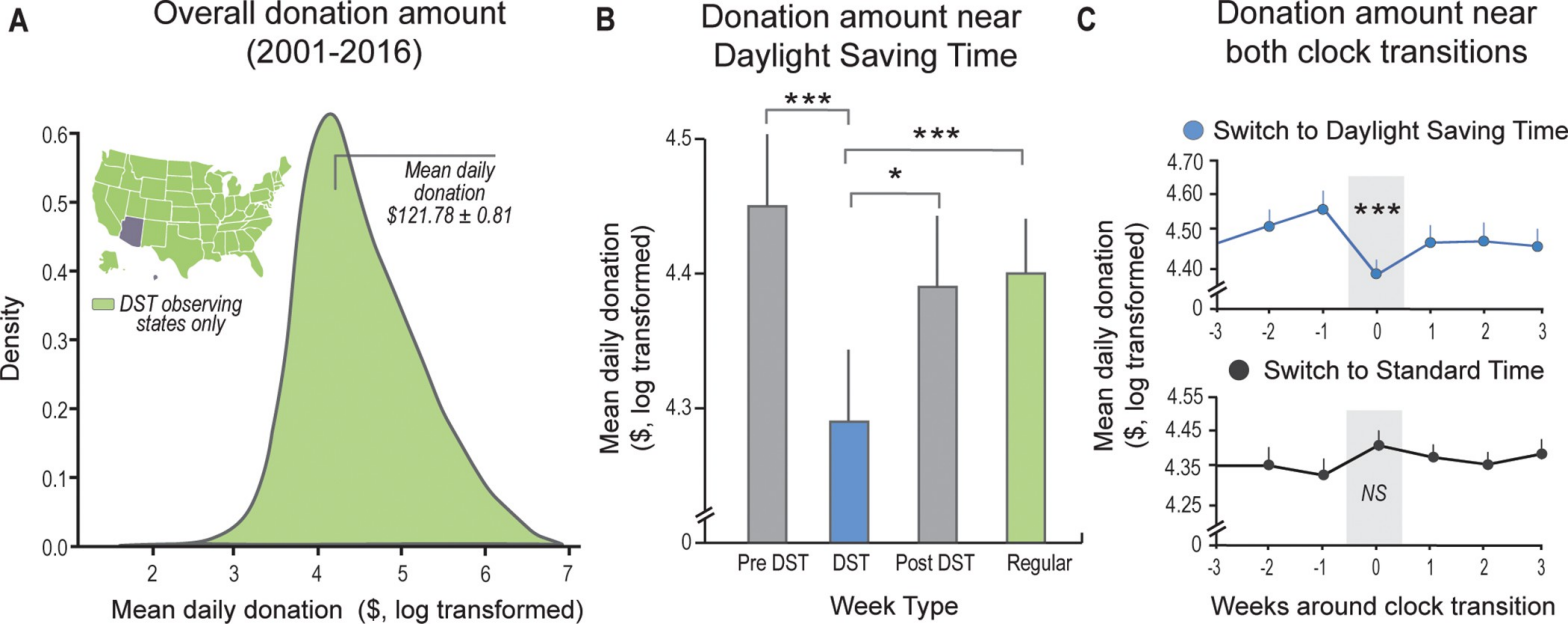
Online donation behavior—Study 3. **(A)** Overall distribution of donation amounts obtained from US states that observe DST, from 2001 to 2016. Light green inset highlights DST-observing states (i.e., excluding Arizona and Hawaii). **(B)** Donation amount was significantly lower in the week of DST transition (light blue) relevant to other weeks in the surrounding months (βDST week = −0.11 ± 0.04, P < 0.005, adjusted for donation day, month, and year, see Methods). **(C)** The reduction in donation amount observed in the weeks around DST (top panel, centered around the third week of March) was not evident in the transition to ST (bottom panel, centered around the second week of November), suggesting that insufficient sleep triggered by the transition to DST uniquely impacts donation behavior. *P < 0.05, ***P < 0.005; error bars reflect standard error of the mean. US base layer map was plotted using the free and open-source Plotly library for python (https://plotly.com/python/maps/). Individual data presented in this figure can be found in S3 Data. DST, daylight saving time; ST, standard time; US, United States.

The Materials and methods section has also been updated to add relevant details related to shared data and code. Please see the updated Materials and methods here:

## Materials and methods

### Online micro-longitudinal study (Study 2)

#### Participants

Study 2 tested whether more modest night-to-night variability in self-reported sleep efficiency and sleep duration predicted day-to-day changes in helping behavior the next day. Unlike the experimental sleep manipulation of Study 1, Study 2 examined sleep variations under free-living conditions. A total of 171 participants (age 36.96 ± 0.73 yr, 41.2% female) signed up for this 4-day study using Amazon Mechanical Turk (MTurk)—a platform where individuals can perform online tasks for a specified reimbursement (here, $4.25–5.75 depending on the final number of daily surveys). Enrollment was restricted to those with IP addresses in the United States, and a prior online MTurk approval rating of 95% or higher. Additional exclusion criteria included a current diagnosis of an Axis 1 psychiatric disorder and/or the confirmed diagnosis of a sleep disorder. Participants were also excluded from further analysis if they completed only one daily survey, which would otherwise have prevented sufficient variability in assessing within-person effects (and see the robustness assessment regarding the main regression using a minimum of 3 or 4 nights of data in Supplementary Note 3 in S1 Text). Furthermore, participants whose sleep logs reported extreme sleep duration values were similarly excluded (less than 3 hr or more than 12 hr). The final sample, therefore, included 136 participants (mean age ± SE = 37.83 ± 0.87y, 41.9% female), yielding a total of 441 valid observations across the study period (See S2 Data, tab for S1 Fig).

#### Study design

Following recruitment, participants were asked to complete validated daily sleep diaries (see Table S6), quantifying their sleep across four consecutive nights. The next day, participants completed an assessment of helping behavior using a shorter form of the helping questionnaire used in Study 1 and described above. The short-form version included 10 social items depicting requests for help, presented in random order, and counterbalanced for familiarity such that requests from strangers and familiar others were equally represented in each daily survey. Each survey day included a different version of the short-form questionnaire depicting different social scenarios. Similar to Study 1, reliability measures for this scale were also strong (Cronbach’s alpha = 0.87 for version 1, 0.85 for version 2, 0.88 for version 3 and 0.9 for version 4). The longitudinal nature of Study 2 further allowed for an examination of test-retest reliability in this sample, which was 0.79 for the first consecutive days of the survey, 0.78 for Days 2–3, and 0.72 for Days 3–4.

To measure helping behavior with respect to prior sleep, the survey was only available online during a specific time window in the morning (until 1 PM local time), and participants were requested to complete the survey as close as possible to their wake-up time. In addition to the key outcome variable of helping behavior, measures of mood were also collected in each daily survey using the short PANAS questionnaire [112] described above. Finally, trait empathy was assessed upon entry to the study using the Interpersonal Reactivity Index (IRI) [82], as in Study 1.

#### Data preprocessing and analysis

Analyses focused *a-priori* on sleep efficiency and sleep duration, given previous work linking both sleep parameters to social and interpersonal functioning [28,30]. Sleep efficiency was measured using participants’ daily sleep diaries, based on the percent of time asleep out of total time in bed (i.e., total time in bed minus sleep latency and time spent awake after sleep onset, mean ± SD = 90.02 ± 9.36%). Sleep duration was calculated as the total time elapsed from sleep onset to wake time minus sleep latency and time spent awake after sleep onset (mean ± SD = 429 ± 68.8 min).

A linear mixed-effects model was calculated to test whether night-to-night variability in sleep efficiency and sleep duration, within participants, predicted day-to-day changes in helping behavior the next day. The *a priori* predictors of sleep efficiency and duration were calculated for both between and within-person effects using person mean centering [113] (see *Statistical analyses* below and S1 Fig). The between-person effect refers to a person’s average over the study period (e.g., mean sleep duration/efficiency across 4 days), while the within-person effect refers to that person’s deviation from their average on a particular day (mean deviation in sleep efficiency = ± 2.03%, mean deviation in sleep duration = ±35.48 minutes or 8.2% of total sleep time). All assessment days were weekdays to avoid concerns of weekend changes in sleep patterns. All linear mixed-effects models were adjusted for age, sex, and survey version.

While the main model focused on sleep duration and efficiency, it is important to note that circadian rhythms and circadian disruption also influence emotional and mood states [114–117]. We, therefore, sought to further empirically explore the contribution of circadian influence to helping behavior. For each participant, the measure of mid-sleep was calculated as the halfway point between sleep onset and sleep offset time during the study period, a marker of habitual circadian phase [118,119]. Similar to the main analysis, mid-sleep was assessed for both between- and within-person effects. Adding these parameters to the main model, there was no significant effect of circadian phase (mid-sleep) on helping behavior (within-person effect β = -0.04 ± 0.03; between-person effect, β = 0.03 ± 0.04, both P > 0.25).

### Online donations database (Study 3)

#### Donation data and analysis

Study 3 tested the prediction that the loss of 1 hr of sleep opportunity (due to Daylight Saving Time) resulted in a real-world, large-scale decrease in helping behavior. Data were obtained from an online database of donations made between the years 2001–2016 in the US, via the DonorsChoose website, a platform that helps raise funds for school projects in the US (e.g., buy books, get supplies for a science project, etc.).

A total of 6,211,956 donations were available for analysis, including information about donor location, the timestamp of each donation, and the project each donation was intended to fund. Donations were excluded from further analyses if they did not include information on date/time or on donor location or were intended for projects that were not eventually funded (e.g., projects that expired before meeting their funding goal or were still not funded at the moment of download). Donations for projects that lasted less than a day were also excluded to allow for more stable predictors of donation behavior over time (including possible effects of sleep).

The main model focused on nationwide donations coming from states that observe DST (i.e., excluding Hawaii and Arizona) totaling 3,871,500 eligible donations (average donation amount $82.27 ± 0.14). For each donation, the following information was calculated and used in the statistical analyses: the day of the week/month/year of the donation and the time of day the donation was made. In accordance with prior reports [41,43,63,120,121], analysis focused on the weekdays following the transition, as both the ST and DST transitions result in sleep consequences lasting up to 5 days before sleep onset and offset times revert, and habitual sleep patterns return [59,122]. Analyses, therefore, focused on a robust window that spanned multiple days of assessment (See Supplementary Note 4 in S1 Text for a secondary analysis focusing on post-transition Monday alone).

Using timestamp data of each donation, analysis tested the hypothesis that during the week of the transition to DST (the weekdays following the second Sunday of March since 2007 or the first Sunday of April before that), the corresponding loss of 1 hr of sleep opportunity would significantly decrease altruistic helping behavior reflected in lower donation amounts. For all analyses, eligible donations were aggregated to a daily average amount across the examined period (2001–2016, total number of observations = 18,454), and subsequently log-transformed before being implemented in a multiple regression model (see *Statistical analyses* below). Donation data were then filtered to exclude extreme outliers (above or below 3 standard deviations from the mean), most of which came from a small number of donors giving very large amounts of money (e.g., more than $100,000 in a single donation), or from days that included extremely high numbers of recorded donations. Following these criteria, a total of 3,420,996 donations, aggregated across 18,034 observations and 5,082 days were utilized in the analyses (average donation amount = $121.78 ± 0.81, see S3 Data, tab for S2 Fig). DST transition analyses examined the 4 weeks before and after the transition to avoid annual seasonal effects on donation amounts (e.g., donation amounts are lower during the summer vacation when school is out, see S2 Fig). This analysis included a subset of 2,925 observations across 816 near transition days.

#### Time of year control analysis

In order to test for nonspecific effects of time of year on donation amounts, three additional models were constructed. The first model examined whether the mere switching of the clock might impact donation behavior irrespective of sleep changes, by focusing on the months surrounding the transition back to ST (the weekdays following the first Sunday of November since 2007 or the last one in October before that). Analysis for this model similarly focused on the 4 weeks before and after the transition to ST as the key predictor of interest and controlling for the same covariates as the main model.

The second model examined possible time of year effects on donation behavior, probing whether the months of March/April when DST transition takes place might impact donation behavior irrespective of changes in sleep. This model focused on the same timeframe as the main model (i.e., the weekdays following the second Sunday of March since 2007 or the first Sunday of April before that) but was now applied to donations coming from Arizona and Hawaii, states that do not observe DST and therefore are unlikely to experience sleep loss during the transition week. Data from Arizona and Hawaii included 76,276 donations made between the years 2001–2016 (average donation amount $70.23± 1.11). As above, this control analysis focused on the 4 weeks before and after the transition to DST as the key predictor of interest, utilizing a subset of 1,675 observations across 553 near transition days (see S3 Data, tab for S3 Fig).

Finally, the last model accounted for time availability effects. Since the day of DST transition is technically a 23-hour day, changes in the available time to make donations, and thus a reduced number of donations, could impact donation amounts, irrespective of changes in sleep. To account for such effects, an additional model was analyzed that controls for the number of daily donations made during the study period, given that the act of donation gifting is indeed subject to time availability irrespective of donation amount. Analyses for this model similarly focused on the 4 weeks before and after the transition to DST/ST as the key predictor of interest, controlling for the same covariates as the main model.

### Helping assessments (Studies 1–3)

#### Helping behavior

The key outcome measures of helping behavior used in Study 1, Study 2, and Study 3, were each designed to offer different but complementary measures of helping, thus allowing us to demonstrate a broader phenotype of prosocial behavior across all studies. The outcome measures taken in Study 1 and 2 assessed participants’ desire to help, using the helping behavior questionnaire, assessing numerous forms of helping deeds and acts common in everyday social life [6,71]. Adding to this, Study 3 examined consequential helping, measuring real-world donation behavior using an online donation database, and thus reflecting a decisive action that directly resulted in the financial giving of help: an altruistic one, since it importantly did not result in (nor depend on) any reciprocal direct financial gain to the donor (a core construct of altruism) [123,124]. As such, and fitting prior studies [20,21,125,126], the assessment of prosocial helping did not incentivize the choice to help others, since acts of altruism typically do not involve reciprocal material exchanges, but instead, are benevolent and uni-directional [123,124].

Thus, Study 1 and Study 2 measure the motivational desire to act altruistically, with the study design choice of a helping behavior questionnaire being motivated by the need to assess a wide selection of prosocial deeds and acts common in everyday life. Broadening the aperture of helping assessment, Study 3 evaluated an altruistic, consequential helping choice, which was the decision to give away money without any incentivized reciprocal benefit.

### Statistical analyses

#### Behavioral results

To test the hypothesis of reduced helping behavior following sleep loss in Study 1, a repeated measure ANOVA was calculated, taking into account familiarity (stranger vs. familiar other) across the different sleep conditions (sleep rested, sleep-deprived). In case of significance, post-hoc tests were computed using paired two-sided T-tests corrected for multiple comparisons using the Bonferroni correction. To control for changes in mood and effort following sleep loss, a multiple linear regression was applied using the difference scores of each variable (sleep deprivation- sleep rested). In this model, the intercept coefficient reflected the sleep-loss effect on helping (i.e., zero denotes no effect), while the mood and effort variables were added as covariates. Associations between social cognition network activity and helping behavior were tested using Pearson’s correlation, with mean activity from the entire *a-priori* network of interest (i.e., not only limited to the activated cluster) to avoid spurious fMRI-behavior correlations [87]. All statistical analyses were conducted using JASP (JASP Team 2021) and the pingouin library implemented in Python [127].

#### Multilevel modeling

Linear mixed-effects models were used to determine associations between sleep and helping behavior across days in Study 2. All multilevel models were adjusted for age, sex, and survey day, with subject identifier defined as a random effect. A person-mean centering was applied to the predictor variables in all models, to disaggregate the between-person and within-person effects [113]. The time-invariant person average and time-varying deviation from an individual’s average were then both included as fixed effects in the multilevel model (between- and within-person components, respectively). To control for outliers in the two key predictors of interest, sleep efficiency and sleep duration values that were ±3STD from the mean were filtered prior to the final analysis. In addition to the main model, two additional models were calculated controlling for changes in 1) mood states (within-person change across days) and innate empathy (a trait variable), and 2) prior helping behavior (a daily variable). These models were identical to the main model in terms of the main predictors and covariates. All multilevel analyses were performed in R [128] using the “lme4”, “lmerTest” and “sjPlot” packages [129–131]. Goodness-of-fit was evaluated with the conditional R^*2*^ [132].

#### Multiple regression

Study 3 implemented a multiple regression model to evaluate the impact of DST transition on donation behavior. The log-transformed daily donation amount in US dollars was set as the outcome variable while the key predictor of interest was ‘week type’ (with levels Monday to Friday before DST, DST week, week after DST, and any other Monday to Friday of the year). All models were adjusted for additional predictors influencing donation likelihood including time of day (using four equal 6-hr bins: morning- 6 am to 11:59 am, afternoon- noon to 5:59 pm, evening- 6 pm to 11:59 pm, and night- midnight to 5:59 am), day of the week, month, year, weekend day (Saturday or Sunday) and holidays (as a binary True/False variable including all major Federal holidays as well as the annual Giving Tuesday which might impact donation amount). For the control analysis of available time to donate, the predictor of number of donations (log-transformed) was also added to the main model. If DST transition lowered donation amount in the five days after switching to DST, a significant coefficient for the level ‘DST week’ was expected compared to all other Monday to Friday in the variable ‘week type’. Similarly, a non-significant coefficient for ‘week type’ was expected in the two control models exploring the transition back to ST as the key predictor or when focusing on donations from states that do not observe DST (i.e., Arizona and Hawaii). All tests of statistical significance were two-sided, and p values less than 0.05 were considered statistically significant. All analyses were performed in R [128] using the “lme4” and “lmerTest” packages.
