# Supplementary figures and images for: Sleep loss leads to the withdrawal of human helping across individuals, groups, and large-scale societies
Source: PLoS Biol. 2022 Aug 23;20(8):e3001733. doi: 10.1371/journal.pbio.3001733 (PMC9398015; doi:10.1371/journal.pbio.3001733)

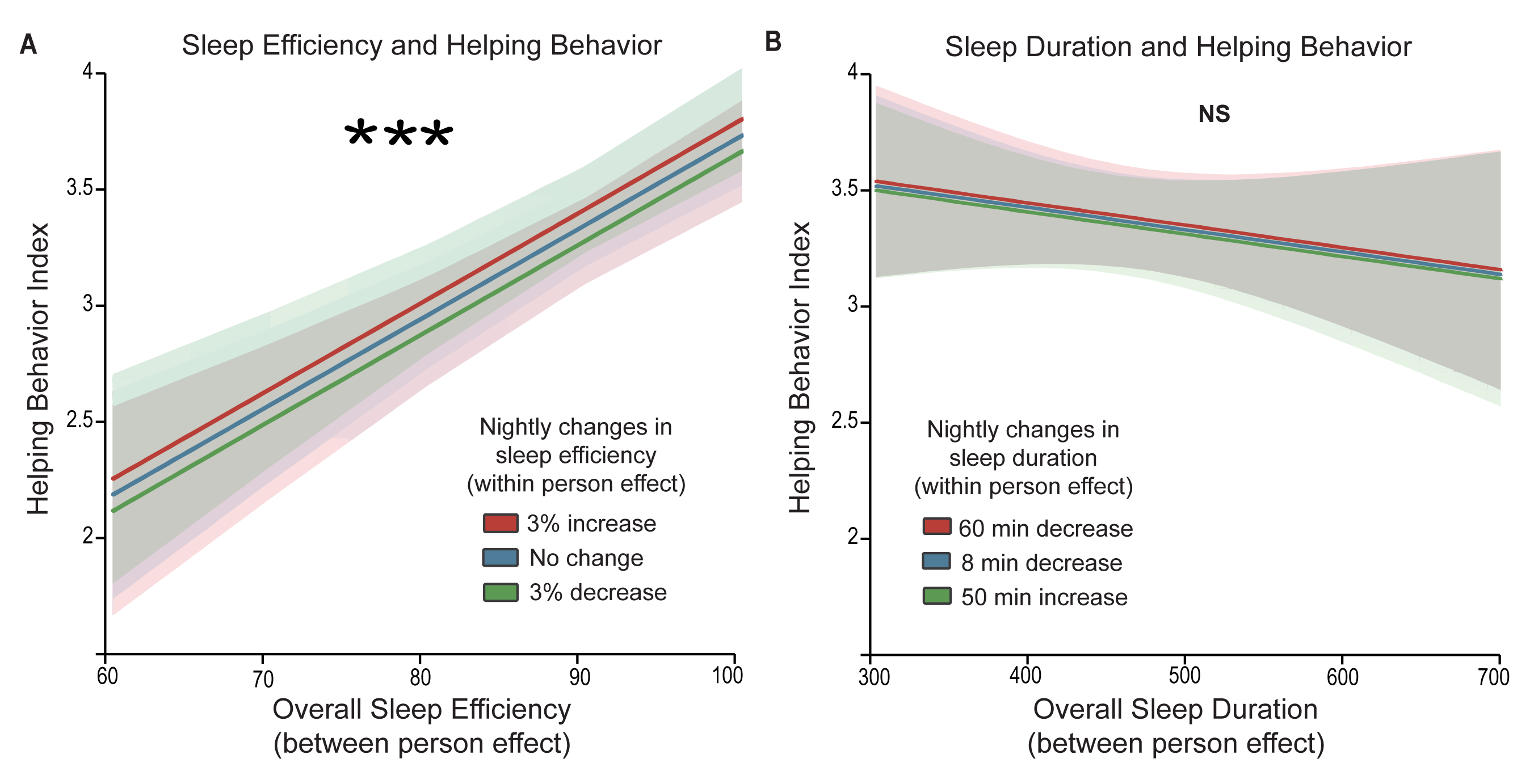

Supplement: S1 Fig — (A) Helping behavior was higher following nights of better sleep quality (within-person effect, β = 0.02 ± 0.01, P < 0.05) as well as in individuals who sleep better overall (between-person effect, β = 0.04 ± 0.01, P < 0.001). (B) No significant effects on helping behavior were found for sleep duration for either nightly changes (within-person effect, β = −0.0002 ± 0.0005, P > 0.6) or changes in sleep duration across participants (between-person effect, β = −0.0007 ± 0.0007, P > 0.3). Model estimates derived from an analysis of 441 observations obtained from 136 individuals across 4 consecutive days. ***P < 0.001. Individual data presented in this figure can be found in S2 Data. (TIF) [file pbio.3001733.s001.tif]

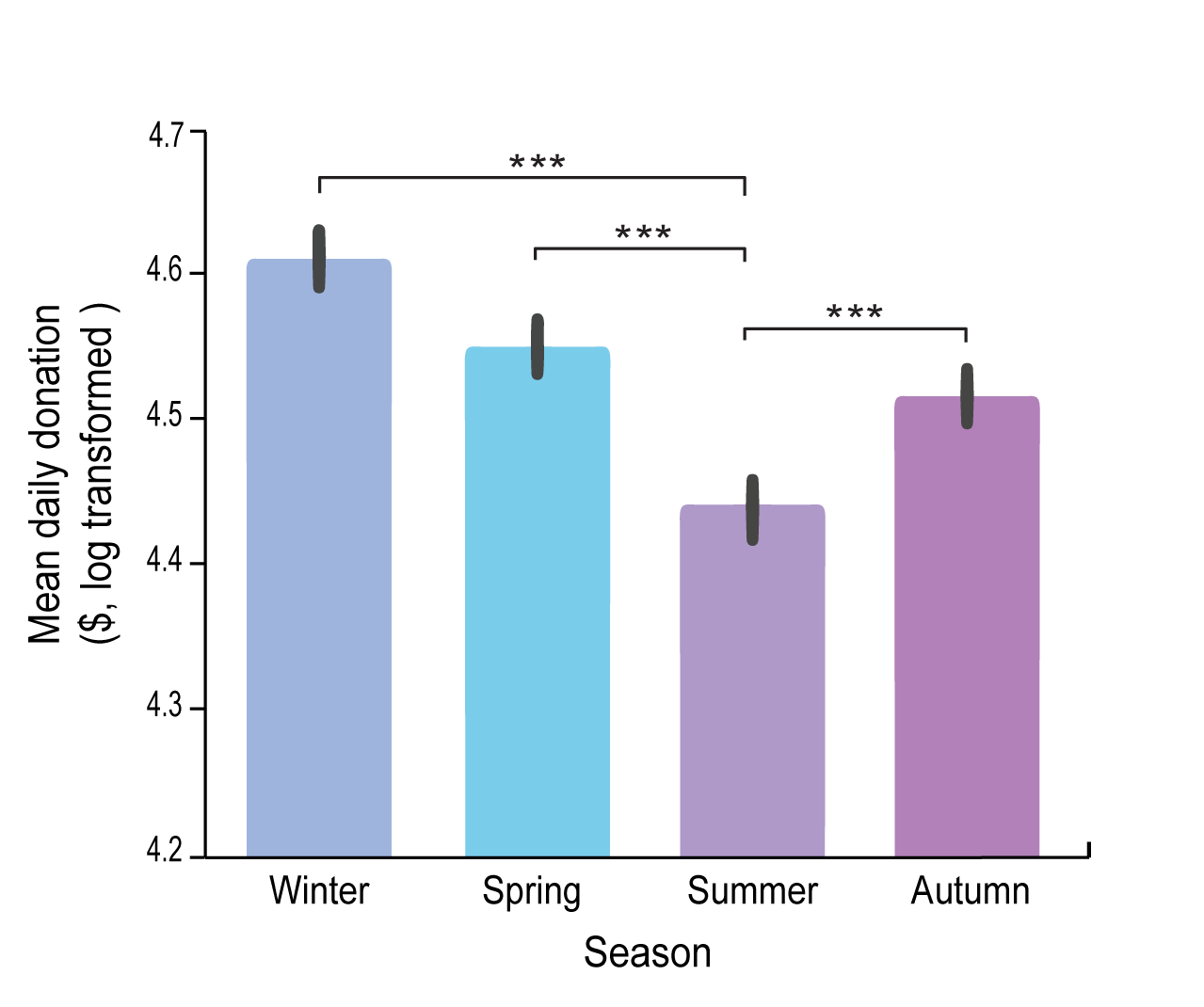

Supplement: S2 Fig — Donation amount was highest in winter and lowest in summer (main effect of season, F (3,67) = 45.17, P < 0.001). Model estimates derived from an analysis of all donations obtained from US states that observe Daylight Saving Time (i.e., excluding Arizona and Hawaii). ***P < 0.001, post hoc t tests corrected for multiple comparisons, error bars denote standard error of the mean. Individual data presented in this figure can be found in S3 Data. (TIF) [file pbio.3001733.s002.tif]

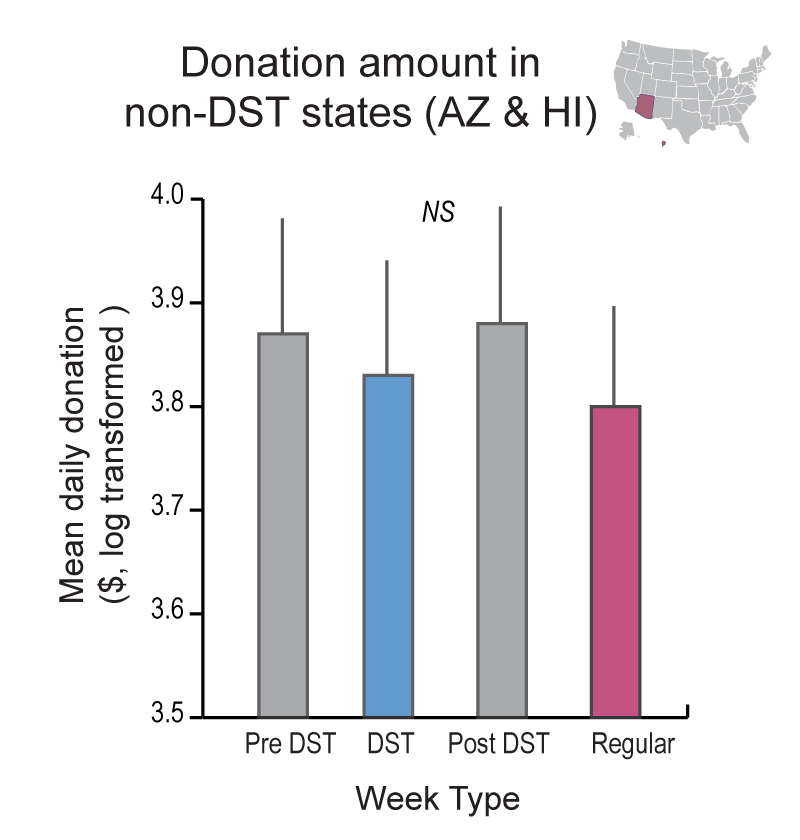

Supplement: S3 Fig — Donation amount was not significantly different in the months surrounding the DST transition in stated that do not experience an actual clock change (β = 0.02 ± 0.08, P > 0.7, including the same covariates as the main model, see Methods). Model estimates derived from an analysis of all donations obtained from US states that do not observe Daylight Saving Time (i.e., Arizona and Hawaii). Error bars denote standard error of the mean. US base layer map was plotted using the free and open-source Plotly library for python (https://plotly.com/python/maps/). Individual data presented in this figure can be found in S3 Data. (TIF) [file pbio.3001733.s003.tif]

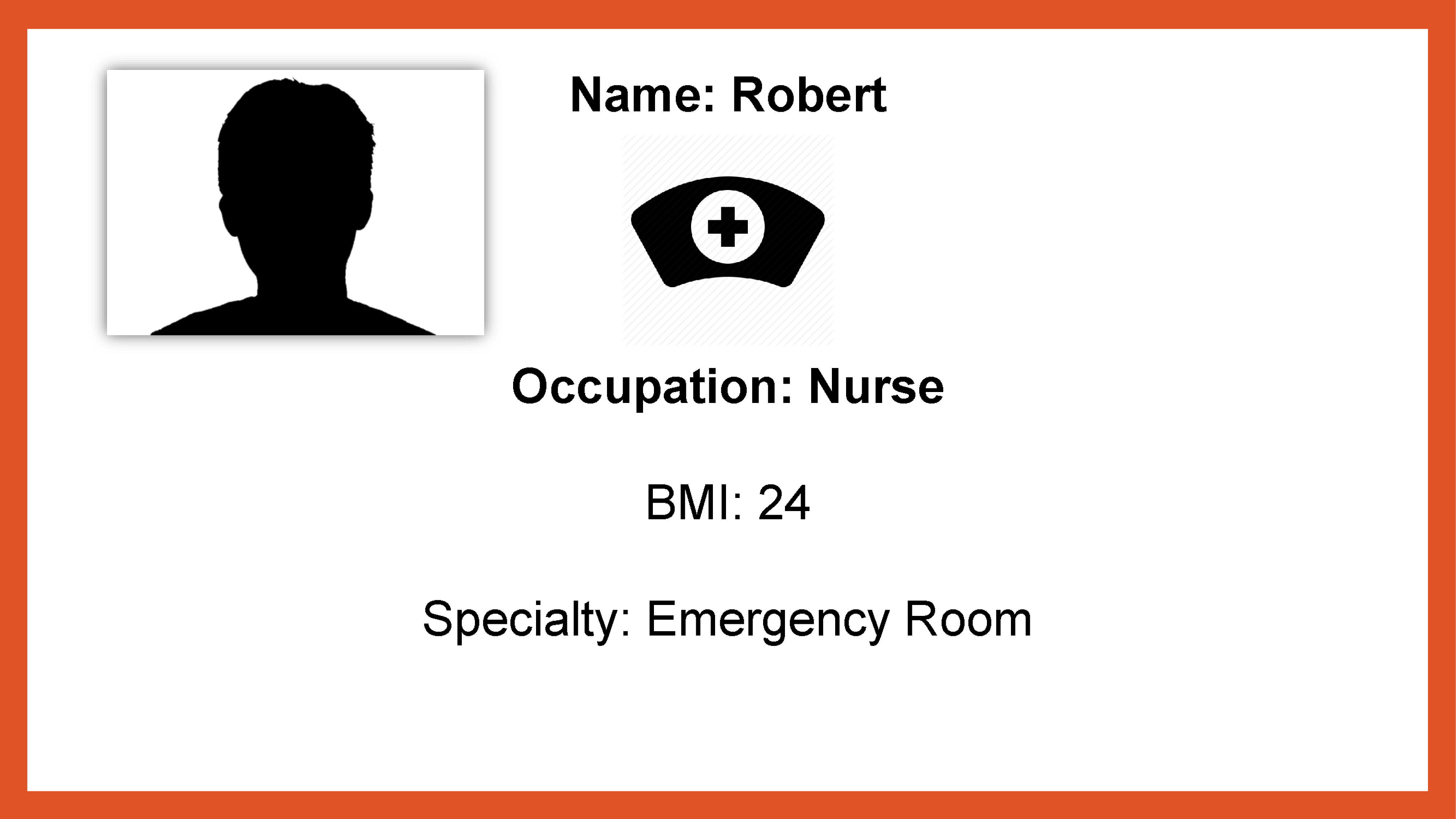

Supplement: S4 Fig — An example of the stimuli used in the social cognition task during fMRI scanning. Each stimulus depicted an individual that participants were asked to assess, including their name, professional details, and a silhouette grayscale image. (TIF) [file pbio.3001733.s004.tif]
