# Supplementary material for: Sleep loss leads to the withdrawal of human helping across individuals, groups, and large-scale societies
Source: PLoS Biol. 2022 Aug 23;20(8):e3001733. doi: 10.1371/journal.pbio.3001733 (PMC9398015; doi:10.1371/journal.pbio.3001733)
Supplement: S1 Text — (DOCX) [file pbio.3001733.s005.docx]

**Supplementary Information**

# Sleep loss leads to the withdrawal of human helping across individuals, groups, and large-scale societies

Eti Ben Simon*, Raphael Vallat, Aubrey Rossi, and Matthew P. Walker*

^*^Correspondence to Eti Ben Simon ([etibens@berkeley.edu](mailto:etibens@berkeley.edu)) and Matthew P. Walker ([mpwalker@berkeley.edu](mailto:mpwalker@berkeley.edu)).

### Supplementary Tables:

Table A: Network Specificity Analysis (Study 1)

Table B: Helping Behavior Questionnaire (Study 1)

Table C: Regions of Interest for fMRI analysis (Study 1)

Table D: Exploratory Whole-Brain Analysis (Study 1)

#### Table E: Polysomnography Sleep Characteristics for the Sleep-Rested Night (Study 1)

Table F: Daily Sleep Logs (Study 2)

### Supplementary Notes:

Note A: Familiarity Effects of the Helping Behavior Questionnaire (Study 1)

Note B: Differential Contrast Weights fMRI Analysis (Study 1)

Note C: Robustness Check for the Analysis of Sleep Efficiency and Helping Behavior (Study 2)

Note D: Monday Only Donation Analysis (Study 3)

#### **Table A: Network Specificity Analysis (Study 1)**

Post-hoc specificity analysis of network activity (Means ± SE) in sleep rested and sleep-deprived sessions using the human vs. object parametric contrast applied in the main analysis. Data was extracted using a validated 7-network parcellation (see main methods for details). None of the activation patterns in these networks showed a significant main effect of sleep deprivation during the social-judgment task (all *P* > 0.1, corrected for multiple comparisons).

|  |  |  |
| --- | --- | --- |
| **FMRI Network** | **Sleep Rested Activity** | **Sleep Deprived Activity** |
| Visual | 0.046 ± 0.15 | -0.332 ± 0.11 |
| Fronto-Parietal | -0.033 ± 0.1 | 0.163 ± 0.07 |
| Limbic | 0.006 ± 0.11 | -0.061 ± 0.12 |
| Dorsal Attention | -0.064 ± 0.09 | -0.168 ± 0.13 |
| Salience | 0.051 ± 0.13 | -0.07 ± 0.09 |
| Somato-Motor | 0.234 ± 0.16 | -0.127 ± 0.16 |

#### **Table B: Helping Behavior Questionnaire (Study 1)**

**Version 1**

| # | Item | Least Helpful Choice | Most helpful Choice |
| --- | --- | --- | --- |
| 1 | If I saw that a stranger on the  sidewalk was somehow injured, I would | Continue walking | Stop and ask how  I could help |
| 2 | If I saw that someone was having trouble starting their car as I am about to leave a parking lot, I would | Leave the parking lot | Offer to help jump start their car battery |
| 3 | I donate money because it benefits others even if it is costly to me | I would only donate if  the effort is not high | The benefit for others is important to me |
| 4 | When I see a student being taken advantage of in a group work, I would | Not involve myself  in the situation | Step in on  their behalf |
| 5 | I would offer my seat on a crowded  bus to a 60 year-old woman | I would get up only  if she asks me to | I would offer my seat immediately |
| 6 | If a stranger asked to use my cell phone to make a call, I would | Say no | Immediately hand them my phone |
| 7 | I would donate money to  the victims of a tsunami | I would not donate | I would definitely donate to them |
| 8 | I would give money to a stranger  who needed it for food | I would not give  any money | I would give  money to him/her |
| 9 | I would voluntarily look after my neighbor's children without being paid | Only for a fair  payment | I would definitely  do so |
| 10 | If I saw a hurt animal on the  side of the road, I would | Continue driving | Immediately stop and call animal services |
| 11 | If an acquaintance tells me  s/he moved houses, I would | Not offer to help | Certainly offer  to help |
| 12 | If my neighbor asks to borrow  my car, I would | Not lend it to  them | Surely lend it  to them |
| 13 | If a student was failing a subject,  I would give him/her private lessons at the cost of my own study time | I will not offer  to do so | I would surely  try to help |
| 14 | When planning my birthday party  I take my guests’ schedules and  wishes into account | I only take my own  schedule into account | I would I always consider my  guests’ schedule |
| 15 | I would help a stranger struggling with her grocery bags to carry them | I would not help | I would definitely help |
| 16 | If a coworker who lives near me  asked me to give her a ride home,  I would | Only if it is not more  than a few minutes  out of my way | Definitely offer her  a ride home |
| 17 | If I was in a hurry to get to  work and someone stopped me  to ask for directions, I would | Ignore them | Stop to help |
| 18 | If a colleague was having their  house fumigated and needed a  place to sleep for the night, I would | Tell them to ask  friends or family first | Offer to let them  stay with me |
| 19 | If I was studying at the library  and my neighbor got up and asked  me to watch their things | I wouldn’t pay attention to their things because I doubt it will be stolen | I would watch their things closely  until they return |
| 20 | If my friend asked for the last  piece of my favorite candy | I would tell them that I prefer to keep it for myself | I would happily give them the last piece |

**Version 2**

| # | Item | Least Helpful Choice | Most helpful Choice |
| --- | --- | --- | --- |
| 1 | I would delay an elevator and hold the door  for a stranger approaching the elevator | Only if I have  a lot of spare time | I would almost  always do so |
| 2 | I would help a classmate who I did not know well with a homework assignment when my knowledge was greater than his or hers. | I would rather not | Sure, if my help is wanted |
| 3 | On my day off I would voluntarily  help children with difficulties at  school do their homework | I would rather do something else | I would certainly help |
| 4 | If an acquaintance spilled a  drink on their shirt right before  a job interview, I would | Continue with my day | Immediately offer  to switch shirts  with him\her |
| 5 | If I had guests stay overnight and  there were not enough beds, I would | Sleep in my own bed  and let the guests  figure out their own sleeping arrangements | Offer my bed to  the guests and  sleep on the floor |
| 6 | Even if I was hungry I would  share my food with others | Only if they ask  me for it | Yes I would offer  it to them |
| 7 | I would stand up for a competent  fellow student to receive a scholarship even if  I would be interested in the scholarship too | Only if I am already  on a scholarship | I would be happy to stand up for  the student |
| 8 | Many people do not donate money,  so I might not need to donate as well | Thus, I do not  donate either | Because of that  I donate |
| 9 | I would donate blood to a teenager  suffering from leukemia | I would not  necessarily do so | Whenever it  is possible |
| 10 | Other people's misfortunes do  not usually disturb me a great deal | I rarely sympathize | I usually feel sorry |
| 11 | When I see a student being  wrongly accused of cheating, I would  step in and clarify the situation | I would rather  do nothing | I would stand up  for him\her |
| 12 | If I prefer action movies to  comedies, I would still see a comedy  if my friends prefer to see that | I would convince them to see the action movie | I would give in and see the comedy |
| 13 | If a friend was sick, I would go to  see him and take care of him  despite the risk of infection | Only if the risk of infection is low | I would always support him |
| 14 | I would allow a stranger with fewer  items to go in front of me  in line at the supermarket | Only If s/he has  one item only | Of course |
| 15 | I would help a stranger  struggling with his\her  luggage to get it onto the bus. | I would not help | I would definitely help |
| 16 | If a coworker had a personal  emergency and asked me to take over  a task they could not complete, I would | Ask them to check with other people first | Gladly take over the task |
| 17 | If I was busy studying for an exam and  a fellow classmate called to ask for  some help on the same topic, I would | Ignore them and continue studying | Suggest they come  over to learn together |
| 18 | If a colleague was planning to visit my hometown and asked to meet for coffee to hear some tips before their visit, I would | Tell them to look up some information online | Definitely meet them for coffee and share  my knowledge |
| 19 | If a classmate asked to borrow my  sleeping bag for a trip they  were planning, I would | Offer they rent a  sleeping bag instead | Definitely lend it  to them |
| 20 | I would donate money to the victims  of an earthquake | I would not  donate to them | I would definitely donate to them |

#### **Table C: Regions of Interest for fMRI analysis (Study 1)**

| **Social Cognition Brain Network ROIs** | | | | | | |
| --- | --- | --- | --- | --- | --- | --- |
| **X** | **Y** | **Z** |  | **Laterality** | **Cluster Size (voxels)** | **Label** |
| -48  56 | -60  -60 | 32  22 |  | Left  Right | 1301  1519 | Temporal-Parietal Junction (TPJ) |
| 2 | -52 | 32 |  | Mid | 1358 | Precuneus |
| 52 | 30 | -8 |  | Right | 240 | Inferior Frontal Gyrus |
| 2 | 44 | -18 |  | Mid | 525 | Ventro-medial PFC |
| 2 | 56 | 22 |  | Mid | 1961 | Dorso-medial PFC |
| -52  56 | 2  -2 | -26  -24 |  | Left  Right | 1741  1665 | Mid Temporal Sulcus |

**MNI coordinates represent the center of mass. All regions of interest derived from a Neurosynth meta-analysis activation map corrected for multiple comparisons at P_FDR_ < 0.01.*

#### **Table D: Exploratory Whole-Brain Analysis (Study 1)**

Exploratory whole-brain analysis showing all peak activations (MNI coordinates, *P* < 0.005 uncorrected, min 5 voxels for paired comparison; sleep rested <> sleep-deprived) of the human vs. object parametric contrast. These *non-a-priori* whole-brain data are provided for fuller descriptive purposes. Cluster size is in voxels; voxel size is 2mm^3^.

| **Sleep Rested > Sleep Deprived** | | | | | | |
| --- | --- | --- | --- | --- | --- | --- |
| **X** | **Y** | **Z** |  | **T** | **Anatomical Label** | **Cluster Size** |
| 23 | -72 | 24 |  | 6.19 | R Superior Occipital Gyrus / Precuneus | 312 |
| -58 | -65 | 10 |  | 4.82 | L TPJ / Mid Temporal Gyrus | 118 |
| 58 | -48 | 20 |  | 4.82 | R TPJ / Superior Temporal Gyrus | 41 |
| 47 | -2 | -22 |  | 4.59 | R Mid Temporal Gyrus | 24 |
| -47 | 1 | -29 |  | 4.85 | L Mid Temporal Gyrus | 19 |
| 19 | -16 | 73 |  | 4.79 | R Superior Frontal | 19 |
| 19 | -30 | 52 |  | 3.75 | R Mid Cingulate | 23 |
| 68 | -44 | 3 |  | 3.67 | R TPJ / Mid Temporal Gyrus | 7 |
| 16 | -41 | 42 |  | 4.28 | Precuneus | 9 |
| 51 | -20 | -8 |  | 3.47 | R Mid Temporal Gyrus | 5 |
| -61 | 1 | -8 |  | 3.51 | L Mid Temporal Gyrus / Temporal Pole | 5 |
| 58 | -16 | -4 |  | 3.60 | R Superior Temporal Gyrus | 7 |
| -8 | 43 | -1 |  | 3.27 | L Anterior Cingulate | 7 |
| -40 | 1 | 10 |  | 3.15 | L Insula | 6 |
| -1 | -27 | 66 |  | 4.42 | L Paracentral Lobule | 13 |
| 44 | 1 | 49 |  | 4.41 | R Precentral | 7 |
| 30 | -48 | 63 |  | 3.83 | R Postcentral | 5 |
| -57 | -34 | 21 |  | 4.03 | L SupraMarginal | 13 |
| 37 | -62 | 31 |  | 3.28 | R Angular Gyrus | 6 |
| 26 | -51 | -50 |  | 3.32 | R Cerebellum | 5 |
| -12 | -72 | -39 |  | 3.46 | L Cerebellum | 11 |

| **Sleep Deprived > Sleep Rested** | | | | | | |
| --- | --- | --- | --- | --- | --- | --- |
| **X** | **Y** | **Z** |  | **T** | **Anatomical Label** | **Cluster Size** |
| 47 | -55 | -15 |  | 3.56 | R Inferior Temporal Gyrus | 10 |
| 33 | -55 | -15 |  | 4.06 | R Fusiform Gyrus | 5 |
| 30 | -83 | -11 |  | 3.37 | R Inferior Occipital Gyrus | 6 |
| 47 | 36 | 13 |  | 3.21 | R Inferior Frontal gyrus | 5 |
| 12 | -46 | 17 |  | 3.15 | R Thalamus | 5 |
| -15 | 1 | 21 |  | 3.41 | L Caudate | 6 |
| -8 | 15 | 28 |  | 3.37 | L Anterior Cingulate | 5 |
| 44 | 15 | 28 |  | 3.63 | R Inferior Frontal Gyrus | 8 |
| -36 | 32 | 28 |  | 4.40 | L Mid Frontal Gyrus | 8 |
| 47 | 32 | 31 |  | 4.37 | R Mid Frontal Gyrus | 24 |

#### **Table E: Polysomnography Sleep Characteristics for the Sleep-Rested Night (Study 1, means ± SE)**

|  | **Time (min)** | **Percentage of total sleep time** |
| --- | --- | --- |
| Sleep latency | 10.62 ± 5.60 |  |
| Total sleep time | 419.18 ± 63.55 |  |
| WASO | 45.21 ± 28.30 |  |
| Sleep Efficiency | 88.16± 6.56 |  |
| NREM stage 1 | 19.08 ± 16.86 | 4.66 ± 4.25 |
| NREM stage 2 | 231.81 ± 54.02 | 55.03 ± 7.56 |
| NREM SWS | 95.85 ± 25.29 | 23.19 ± 6.19 |
| REM | 72.44 ± 27.65 | 17.11 ± 5.26 |

*WASO, Wake After Sleep Onset; NREM, Non Rapid Eye Movement sleep; SWS, Slow-Wave Sleep (NREM stages 3 and 4); REM, Rapid Eye Movement sleep*

#### **Table F: Daily Sleep Logs (Study 2)**

Sleep diary questions for Study 2. Participants completed the sleep diaries across four consecutive nights of habitual sleep. Questions were presented in random order in each daily survey.

| **Daily Sleep Diary** | | |
| --- | --- | --- |
| Q1. |  | What time did you go to bed last night? |
| Q2. |  | How long did it take you to fall asleep last night? |
| Q3. |  | What time did you wake up this morning? |
| Q4. |  | How many times did you wake up during the night? |
| Q5. |  | If you woke up during the night, how long did it take you to get back to sleep? |

#### **Note A: Familiarity Effects of the Helping Behavior Questionnaire (Study 1)**

#### The helping behavior questionnaire was equally divided between helping requests concerning strangers versus personally familiar others. Focusing on this dichotomy, there was no main effect of strangers vs. familiar others in the main ANOVA analysis (F_(1,22)_ = 1.76, η_p_^2^ = 0.074, *P* = 0.198). When assessing the difference between strangers and familiar others separately, there was no significant difference, in either the SD condition (familiar other = 3.66 ± 0.14, strangers = 3.52 ± 0.13, *P* = 0.1), nor the SR (familiar other = 3.91 ± 0.12, strangers = 3.85 ± 0.12, *P* = 0.52).

#### **Note B:** **Differential Contrast Weights fMRI Analysis (Study 1)**

The in scanner social judgment paradigm contained two repeatable versions, each including a different set of 80 information cards (48 humans and 32 objects). To ensure that the increased sampling of social trials did not bias the main result, new contrasts were constructed for each participant, using differential weights that compensated for the category imbalance (applying a 1:1.5 ratio for human and object trials respectively). This analysis, therefore, examines the changes in brain activity triggered by lack of sleep when both human and object trials are equally balanced. Similar to the main analysis, results revealed a significant reduction in social-cognition brain activity in the sleep deprivation, relative to the sleep rested condition (mean sleep-deprivation change = −0.59 ± 0.28, *P* = 0.045, d = 0.62). Such findings would suggest that the differences in brain activity associated with insufficient sleep are not best accounted for by differences in the number of trial-category types.

#### **Note C: Robustness Check for the Analysis of Sleep Efficiency and Helping Behavior (Study 2)**

#### The main regression model in Study 2 used a minimum of 2 survey nights as inclusion criteria to allow for robust statistical analysis of both within- and between-person changes in sleep. To confirm the robustness of this 2-night minimum (rather than selection of 3 or 4 nights), two supplemental regression models were repeated, one with a cutoff threshold of >3 nights, and one with a cutoff threshold of 4 nights (n=381 and 286 observations respectively). The results of these two modified regression models remained supportive of the experimental hypothesis: worse sleep efficiency resulted in significant impairment in helping: 3 minimum nights - β = 0.04 ±0.007, 4 minimum nights - β = 0.04 ±0.01, both *P* < 0.001, the between-person effect, and a trend for the within-person effect: β = 0.016 ±0.009 (3 nights), *P* = 0.066, β = 0.017 ±0.01, *P* = 0.098 (4 nights).

#### **Note D: Monday Only Donation Analysis (Study 3)**

In addition to the main DST analysis window that spanned multiple days, we analyzed donation amount focusing on the Monday of the transition relative to the two control weeks before and after (n = 147 and 149 observations for the DST/ST transitions respectively). Results reveal a similar impact of lower donation amount on the Monday of the transition relative to the week prior, though likely due to lowered power to test the hypothesis, the effect was only marginally significant (β_DST Monday_ = -0.19 ± 0.11, *P* = 0.09, controlling for donation time and year). For the ST transition there was no significant effect of the Monday following the transition relative to the week prior, similar to the main analysis (β_ST Monday_ = -0.03 ± 0.12, *P* = 0.82, controlling for donation time and year).
